# Systematic age‐, organ‐, and diet‐associated ionome remodeling and the development of ionomic aging clocks

**DOI:** 10.1111/acel.13119

**Published:** 2020-04-23

**Authors:** Bohan Zhang, Dmitriy I. Podolskiy, Marco Mariotti, Javier Seravalli, Vadim N. Gladyshev

**Affiliations:** ^1^ Division of Genetics Department of Medicine Brigham and Women's Hospital and Harvard Medical School Boston MA USA; ^2^ Redox Biology Center and Department of Biochemistry University of Nebraska‐Lincoln Lincoln NE USA

**Keywords:** aging, biomarkers of aging, calorie restriction, chemical elements, ionome

## Abstract

Aging involves coordinated yet distinct changes in organs and systems throughout life, including changes in essential trace elements. However, how aging affects tissue element composition (ionome) and how these changes lead to dysfunction and disease remain unclear. Here, we quantified changes in the ionome across eight organs and 16 age groups of mice. This global profiling revealed novel interactions between elements at the level of tissue, age, and diet, and allowed us to achieve a broader, organismal view of the aging process. We found that while the entire ionome steadily transitions along the young‐to‐old trajectory, individual organs are characterized by distinct element changes. The ionome of mice on calorie restriction (CR) moved along a similar but shifted trajectory, pointing that at the organismal level this dietary regimen changes metabolism in order to slow down aging. However, in some tissues CR mimicked a younger state of control mice. Even though some elements changed with age differently in different tissues, in general aging was characterized by the reduced levels of elements as well as their increased variance. The dataset we prepared also allowed to develop organ‐specific, ionome‐based markers of aging that could help monitor the rate of aging. In some tissues, these markers reported the lifespan‐extending effect of CR. These aging biomarkers have the potential to become an accessible tool to test the age‐modulating effects of interventions.

## Graphical abstract

The study revealed systematic organ‐ and diet‐associated ionome remodeling with age. It also reports the ionomic aging clocks that can be used to assess the biological age of mice.

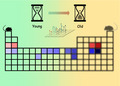

## 1 INTRODUCTION

Aging is associated with systemic deleterious changes at all levels, from molecular to organismal, leading to a gradual decline in function. During aging, organisms accumulate diverse forms of damage and other deleterious changes, ranging from the consequences of errors in intrinsic biological processes to the effects of extrinsic factors coming from diet and environment (Gladyshev, 2016; Lopez‐Otin, Blasco, Partridge, Serrano, & Kroemer, 2013). These intrinsic and extrinsic risk factors interfere with normal functions, causing alterations to the genome, epigenome, proteome, transcriptome, and metabolome (Booth & Brunet, 2016).

Age‐related changes include changes in the levels of chemical elements (Meplan, 2011). Human body is composed of about 60 elements, about one third of which have known biological functions (Chellan & Sadler, 2015). Some of these elements are macroelements, that is, elements characterized by high abundance. They form the basic structures of molecules and tissues. For example, Na and K contribute to the electric potential produced by nerve cells, which is key to all neuroactivities (Chellan & Sadler, 2015). Ca is not only a key component of bones and teeth, but also regulates contractions of cardiac muscle cells through its cation form (Vaughan‐Jones, 1986). Mg plays a role in regulating cell cycle, specifically in the replication, transcription, and translation (Walker, 1986). S is a component of two amino acids and numerous cofactors (Kessler, 2006). In addition to the key role P plays in nucleic acids, it is a major component of the bones.

Some elements in the body are known as trace elements. Despite their lower levels, these elements (many of which are transition metals) play important roles in vital biological processes. For example, Mn is well known as a critical component of Mn‐superoxide dismutase and several other proteins (Avila, Puntel, & Aschner, 2013). Fe acts through hundreds of Fe‐containing proteins, such as the famous heme‐containing protein acting as the carrier of oxygen in the body. Zn is also a component of hundreds of proteins and is central to the regulation of development and wound healing, as well as the immune system and reproductive functions (Oteiza & Mackenzie, 2005; Stefanidou, Maravelias, Dona, & Spiliopoulou, 2006). Cu acts as a cofactor of redox enzymes and contributes to a set of biological processes such as cardiovascular development (Bost et al., 2016). Co is actively involved in neuroprotection and hematopoietic systems through the function of vitamin B12. Mo is a key part of the MoCo cofactor, a critical contributor to neurological functions (Hänsch & Mendel, 2009). Finally, as a functional component of selenoproteins, Se plays important roles in redox control, thyroid function, and other biological processes (Labunskyy, Hatfield, & Gladyshev, 2014). In addition to these essential elements, there are elements in the body that do not have known biological functions, such as As and Cd. Although As has been utilized for treating human diseases such as leukemia, its use is related to its toxic properties (Shen et al., 1997).

The balanced element composition in the body is closely related to its health status, whereas an altered homeostasis of elements may lead to diseases, some of which resemble functional decline related to aging. For example, a shift in the balance of elements, especially metals, may predispose to neurodegenerative diseases. Overexpression of amyloid beta, a protein implicated in the Alzheimer's disease, leads to Mn accumulation inside the brain, and excess Fe and Cu also accelerate the progression of this disease. Parkinson's disease, in addition, was reported to be related to altered Mn homeostasis and elevated Fe levels, and the Down Syndrome was associated with altered Zn levels (Fraga, 2005; Gaeta & Hider, 2005). Furthermore, changes in Cu were linked with diabetes and cardiovascular diseases, both of which are age‐related diseases (Uriu‐Adams & Keen, 2005). On the other hand, the effect of aging on the composition of elements is not well understood.

In the current study, we sought to better understand aging by analyzing changes in element composition throughout lifespan. We quantified the ionome (19 element isotopes) of eight organs of mice aged 3–35 months old. We observed that the distribution of elements reflects their organ of origin and that age‐related changes in this distribution are gradual. We also found clear differences between control mice and mice subjected to calorie restriction (CR) at multiple levels. In addition, we explored the possibility of building organ‐specific biomarkers of aging based on organ ionomes and developed a series of clocks that could track the aging process and report the effect of CR. These findings offer a better understanding of aging from the perspective of element composition and provide a convenient tool that may be applied to assess the biological age of animals.

## 2 RESULTS

### 2.1 Organ ionomes are stable throughout life

**Figure 1:**
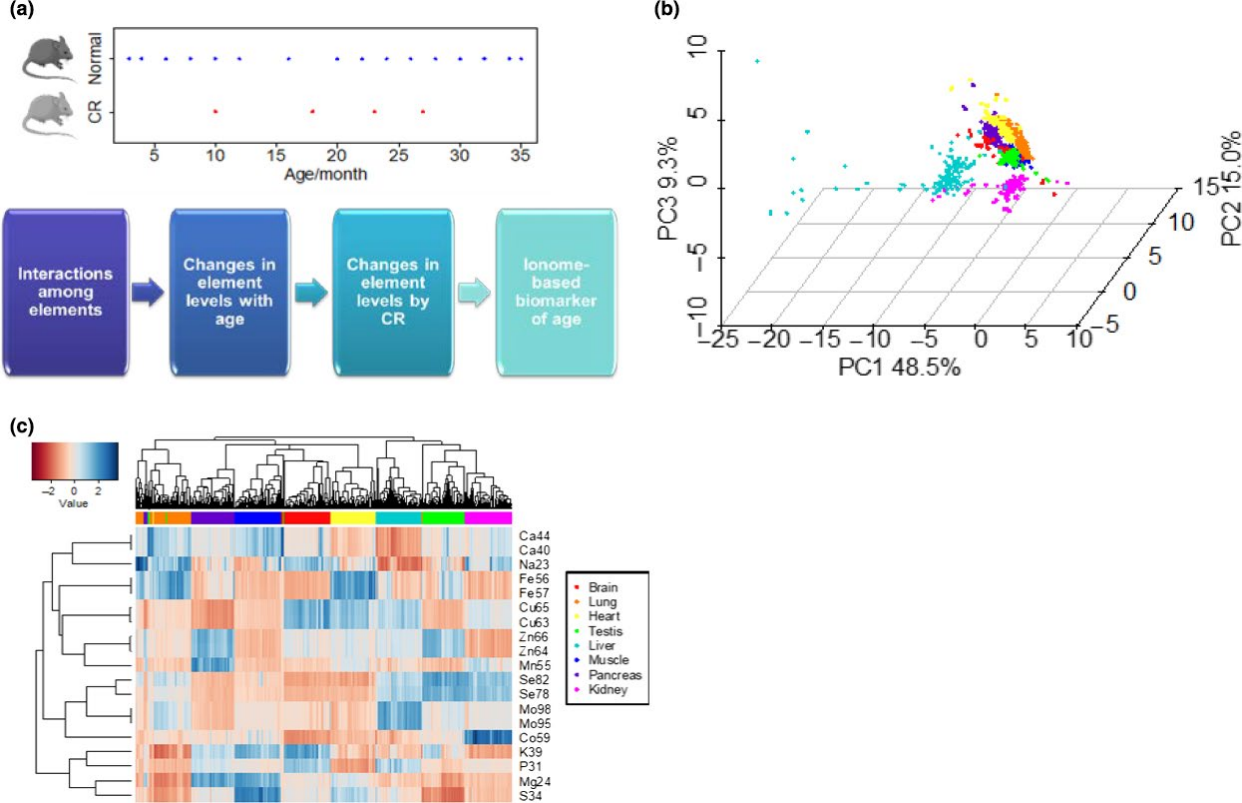
Overview of mouse organ ionomes. (a) Age distribution of mouse samples and schematic of analyses in the study. Sixteen age groups (3–36 months old) of mice on a standard diet and four age groups (10–27 months old) of mice on calorie restriction were analyzed. All mice were C57BL/6 animals. (b) Principal component analysis of samples. Organ origin is shown with different colors. Replicates are presented as individual points. (c) Heatmap view of samples and elements. Each row represents one element or isotope. Each column represents one particular biological sample. Elements with contents lower than noise in certain tissues (e.g., Cd and As) are shown in Figure S2. Clustering was performed using complete‐linkage method with Euclidean distance measure. The same color scheme of organ of origin (shown on the right) is used for panels (b) and (c)

We prepared a large‐scale dataset of ionome profiles of C57BL/6 mice representing eight organs (brain, lung, heart, testis, liver, muscle, pancreas, and kidney) and 16 age groups ranging from young adults (3 months old) to very old mice (35 months old), with up to 4–5 biological replicates per age group (Figure 1a). After filtering, the resulting dataset included 1,271 tissue measurements. We assayed 20 chemical elements, including 10 elements that were assessed by two different isotopes, by using inductively coupled plasma‐mass spectrometry (ICP‐MS) and could reliably quantify 19 isotopes representing 13 elements (Na, Mg, Ca, P, S, K, Mn, Fe, Co, Cu, Zn, Se, and Mo) across the entire mouse lifespan. Additional elements (As, Cd) were also quantified and analyzed, but were not detected in all tissues. Principal component analysis (PCA) revealed distinct sample clustering by the first three principal components (Figure 1b). The samples clustered by organ of origin, indicating high stability of element composition of organs throughout adult life. This observation is consistent with a previous ionomic analysis of young adult males across different species of mammals (Ma et al., 2015). Interestingly, liver samples exhibited a wider distribution, implying a higher diversity in the ionome structure of this organ. We examined these outliers in more detail and found that almost all of them belonged to the tissues of mice aged 30–35 months, suggesting that the liver is more prone to the disrupted elemental composition with age than other tissues examined.

Hierarchical clustering also resulted in the samples being grouped by their organ of origin (Figure 1c). Different isotopes of the same element showed very tight clustering, as they are similarly utilized by an organism. The two analyzed Se isotopes, Se^78^ and Se^82^, showed a slight variation in the lung, although the basis of this effect is unclear. We examined similarity in element profiles across organs and found that Na and Ca clustered together, whereas Mg and K clustered with S and P. Also, most transition metals clustered together, except for Mo and Co, which were closer to Se. They showed higher levels in the kidney and lower in the brain and heart. Ca and Mg were elevated in the muscle, which agrees with their reported distribution in the body (Jahnen‐Dechent & Ketteler, 2012).

### 2.2 Interactions among elements

**Figure 2:**
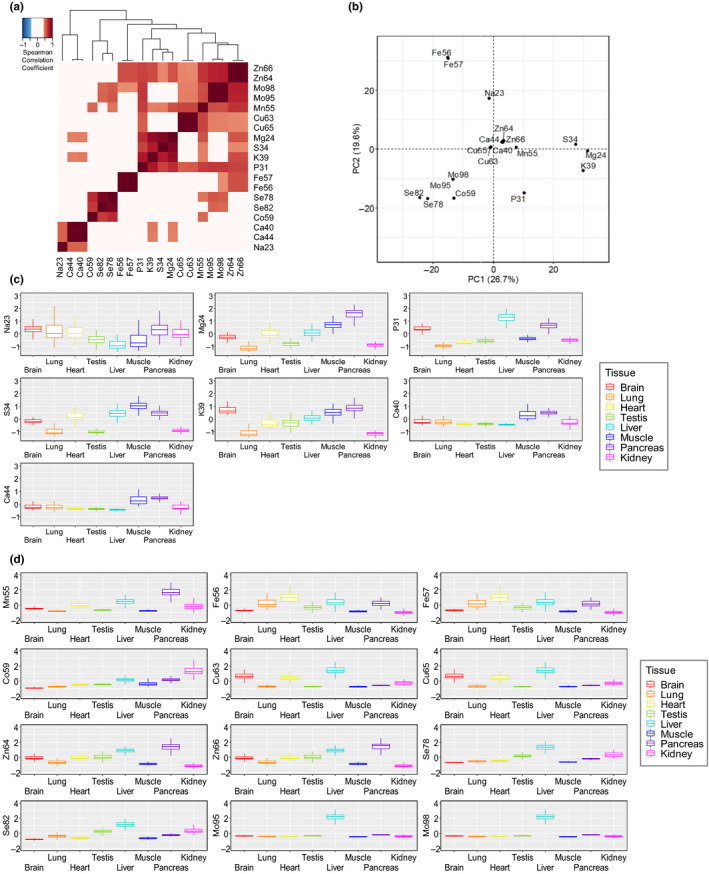
Features of elemental composition across organs. (a) Correlation coefficient matrix of elements across eight organs. Coefficients >0.4 or <−0.4 are highlighted in color. (b) Principal component analysis of elements. The 19 isotopes are projected on the first two PCs. (c) Macroelement composition of different organs. Comparison between the scaled value of element content is performed for eight organs. (d) Trace element composition of different organs. Comparison between the scaled value of element content is performed for eight organs

To examine associations among elements across lifespan, we calculated their Spearman's correlation coefficients for tissues and age groups (Figure 2a). As expected, isotopes of the same element strongly correlated, exhibiting coefficients above 0.9. We previously found that Fe, Co, Mn, and Mo form a cluster across 26 species of mammals (Ma et al., 2015). In contrast, the macroelements Mg, S, P, and K formed a cluster across the mouse lifespan (Figure 2a). This observation may be related to the high levels of these elements in mammalian tissues. In addition, K and Mg were highly correlated to Ca, consistent with their involvement in common regulatory functions.

The trace elements Mn, Cu, Zn, and Mo formed another cluster. We also observed a cluster of Ca and Na. This is consistent with the pervasive sodium–calcium exchange in biology. Excitable cells (mostly neurons) use a sodium–calcium exchanger to export the Ca ion when taking in the Na ion, preventing Ca accumulation. Principal component analysis offered further information on the correlation matrix of elements (Figure 2b). Interestingly, Mn, Ca, Cu, and Zn clustered in the middle of the plot. These elements are in the same period on the periodic table, and they all are characterized by biologically functional divalent states, even though they have different ionic radii, suggesting that the chemical properties of elements may influence their utilization.

We further focused on the distribution of elements across tissues (Figure 2c,d; Figure S3). Liver, kidney, and testis showed the highest levels of Se. Kidney exhibited a large range of element levels, and surprisingly, it also had the lowest Zn and Fe values. We also found that testis had high levels of Zn, supporting its a role in sperm function (Fallah, Mohammad‐Hasani, & Colagar, 2018).

### 2.3 Age‐associated ionome remodeling

**Figure 3:**
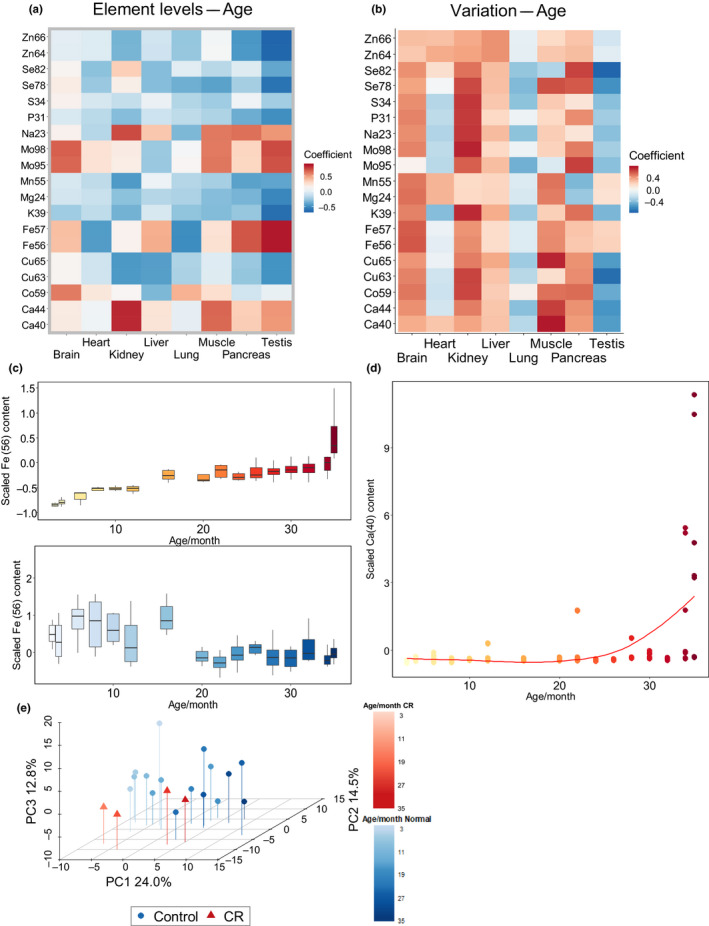
Changes in element levels with age. (a) Spearman's correlation coefficient between age and element levels. (b) Spearman's correlation coefficient between age and element coefficient of variation. (c) Distinct age‐related changes in iron levels in the testis and lung. A complete representation of element changes is in Figure S4. (d) Age‐related increase in calcium levels in the testis. Calcium increases sharply in the oldest ages. (e) Principal component analysis of samples based on age and diet. Transition across age is indicated with increased color intensity: Blue circles indicate animals on a standard diet, and red triangles indicate animals on a calorie restriction (CR) diet. The figure shows percent variation explained by the first three components

We sought to determine how the element levels change across tissues with age (Figure 3a). Most elements (eight out of 13) had a negative correlation with age in all or almost all organs analyzed, consistent with an acquired systemic deficiency of these elements (Zn, Cu, Se, Mn, Mg, P, S, K) in old age. The remaining five elements showed contrasting patterns in different tissues or were increased with age; for example, we observed elevated Ca in kidney and muscle, Fe in pancreas and testis, and Mo in brain and testis. We also examined how the variability in element levels changes with age by analyzing the coefficient of variation (Figure 3b). We observed a pattern, wherein most elements in most tissues were characterized by increased variation with age. This is consistent with disrupted homeostasis of tissues with age. Notable exceptions were the lung and testis.

We examined in more detail the elements that exhibited contrasting age‐related trends (Figure 3c). The Fe levels in the testis as well their variability increased with age, whereas this element showed a totally different pattern in the lung. The main source of Fe in the lung is the serum, so a decrease in Fe may be the sign of a reduced blood flow in this organ. A similar pattern was observed in the testis. This is consistent with oxidative damage in response to high Fe (Turner & Lysiak, 2008). In addition to the elements steadily increasing with age, we noticed elevated Ca in the brain and testis specifically in the very old mice (Figure 3d). In the testis, this dramatic increase was only observed in the oldest mice (34–35 months old) and it also varied dramatically within these age groups. The data suggest that massive calcification may occur at very advanced ages.

Because the elements such as Ca showed sharp increases in some tissues in the oldest ages, we were interested to determine whether age‐associated changes in element levels occur gradually or are subject to sudden shifts at a certain age. To examine this question, we carried out PCA of samples grouped by their age. Interestingly, one of the principal components was defined by mouse age (Figure 3e), showing that the entire ionome moved along a particular trajectory, with gradual changes in element levels with age (Figure S6). Strikingly, CR mice showed a trajectory in the same direction, which was shifted along another principal component. We interpret these data as that, rather than slowing down the aging process, at the organismal level CR remodels metabolism, so that the organism ages along a different (shifted) trajectory that is still going in the same direction.

### 2.4 Organ‐ and element‐specific effects of caloric restriction

**Figure 4:**
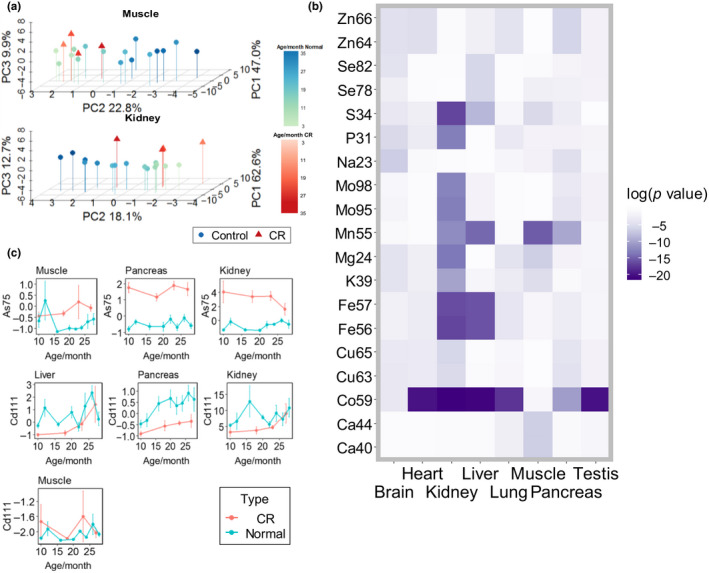
Effect of calorie restriction on the ionome. (a) Principal component analysis of muscle and kidney samples according to age and diet. Older groups are shown by darker color. Blue: animals on a standard diet. Green: animals on a CR diet. Percent variation explained by the first three components is shown in the Figure. Principal component analysis of other organs is in Figure S5. (b) Heatmap of *p* values calculated according to Wilcoxon sign‐ranked test between control and calorie restriction (CR) groups and adjusted with Benjamini and Hochberg method. (c) Line plots of As and Cd content in the muscle, pancreas, and kidney. The *y* axis shows the scaled value of the element content, and the *x* axis shows the age

Calorie restriction is one of best characterized longevity interventions in mice. Similar to the analysis of the organismal ionome (Figure 3e), PCA within individual organs (lung, heart, pancreas, muscle, and kidney) revealed an aging trajectory (Figure 4a; Figures S5 and S6). Interestingly, the trajectories of CR and control samples were aligned in the muscle and kidney. For these two organs, CR mice corresponded to younger control mice, suggesting that in these organs the CR effect may resemble a shift to younger state in terms of element composition.

To further visualize general differences in element content between the two dietary groups, we applied a Wilcoxon signed‐rank test to CR mice and animals on a standard diet and generated a heatmap of significance values (Figure 4b). The most striking element was Co, with significant differences in six out of the eight organs. In addition, Mn showed significant differences in the liver, muscle, kidney, and pancreas. Among the tissues, the kidney was most affected by CR, including both macroelements and trace elements. We also investigated changes in elements with age in CR mice and found that elements such as Mo in the liver showed a different trend in CR and control mice (Figure S6).

### 2.5 Caloric restriction elevates arsenic and decreases cadmium

There were several elements below the detection limit in some tissues, precluding global analyses of these elements. These included As and Cd, which are thought to be toxic and lack biological functions in mammals. We examined these elements in the organs where they could be quantified, namely muscle, liver, pancreas, and kidney. Surprisingly, in organs with detectable As, this element was elevated in CR mice compared to controls, whereas Cd showed decreased levels in CR mice in all tissues except the muscle (Figure 4c). The kidney showed the most significant difference in As between CR and control mice, while the pancreas showed this for Cd. The decreased Cd in CR mice may be related to the reduced amount of food consumed over time, but the basis for the elevated As in CR mice is currently unclear.

### 2.6 Accurate organ‐specific ionomic biomarkers of age

We tested the possibility of using our ionome dataset to quantify the aging process in mice. By applying elastic net regression of ionome data on chronological age of mice separately for eight individual organs, we developed the biological markers of age for each of these organs. Each developed marker represented a weighted average abundance of different elements in the ionome which correlated most strongly with age (Table 1). The established weighted average abundances in the ionomes representing the brain, heart, liver, lung, and muscle did not correlate with age sufficiently well to have predictive power for biological age evaluation on test samples (*R* ^2^ = .64, .58, .01, .50, .37, respectively). For these tissues, we were also unable to discriminate between the mice on standard and CR diets.

**Table 1:** Characteristics of the ionomic biomarkers of age

Tissue	*R* ^2^ (marker age vs. chronological age)	St. dev. error, months	MAE, months	No. of elements in the marker (out of 30)
Brain	.64	3.51	2.58	7
Heart	.58	4.63	3.48	8
Kidney	.84	2.21	1.85	12
Liver	~0	12.05	10.94	0
Lung	.50	4.44	3.16	10
Muscle	.37	5.07	4.03	3
Pancreas	.82	2.19	1.75	15
Testis	.85	0.77	0.68	16

Note

The developed ionomic biomarkers of age are more precise for the tissues shown in green (kidney, pancreas, testis), less precise for the tissues shown in blue (brain, heart, lung), and inaccurate for the tissues shown in red and yellow (muscle, liver).

Abbreviation: MAE, mean absolute error.

**Figure 5:**
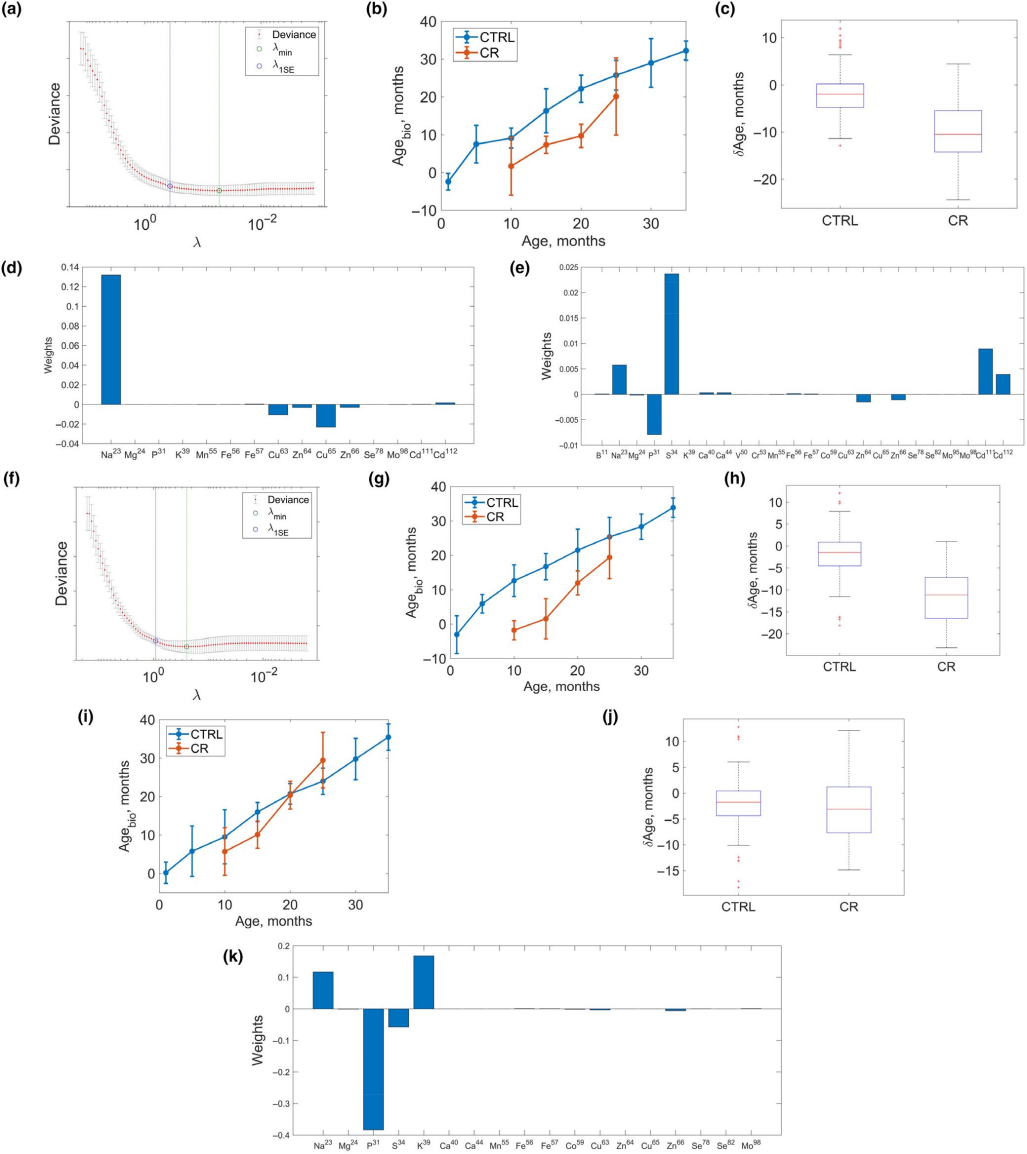
Ionome‐based biomarkers of age in mice. (a) Building the ionome marker of age for mouse kidney. Marker deviance is a function of Tikhonov regularization parameter *λ* (see Methods).* λ*_min_ corresponds to the minimum of deviance as a function of *λ*. Deviance itself is identified with error which can be determined performing cross‐validation. The value *λ*_1_ *_SE_* corresponds to the deviance 1 standard error away from *λ*_min_. (b) Pace of the ionome biological marker of age based on mouse kidney. Biological age of control samples is shown in blue and calorie restriction (CR) samples in red. (c) Difference $\delta \text{Age}={\text{Age}}_{\text{biological}}-{\text{Age}}_{\text{chronological}}$ between chronological ages of mice from which the samples were collected and their biological ages as determined by the kidney ionome clock. The biological age of CR mice is lower on average by 10 months than that of control mice, *p* = .03 (one‐sided two sample *t* test). (d) Weights of different elements in the linear combination representing the kidney ionome marker of age. (e) The same for the pancreas ionome clock. (f) Building the ionome marker of age for mouse pancreas, deviance is a function of Tikhonov regularization parameter *λ*. (g) Pace of the pancreas ionome biological marker of age. The biological age of control samples as a function of their chronological age is shown in blue and that of CR mice in red. (h) Same as Figure 5c, but for the pancreas. The biological age of CR mice was lower on average by 8 months, *p* = .01 (one‐sided two sample *t* test). (i) Same as (b) and (g), but for the testis. (j) Same as (c) and (h), but for the testis. (k) Same as (d) and (e), but for the testis

However, for kidney (Figure 5a–d), pancreas (Figure 5e–h), and testis (Figure 5i–k) elastic regression resulted in the biomarkers with strong correlation with age (*R* ^2^ = .84, .82, .85 between average element abundance and chronological age of samples), with mean average error in age identification for test samples of ~1.85, ~1.75, and ~0.68 months for the kidney, pancreas, and testis, respectively (Table 1; mean absolute error defined according to the prescription $\text{MAE}=\frac{\sum_{i}\left|{\text{Age}}_{\text{ion}}-{\text{Age}}_{\text{chron}}\right|}{n}$, where Age_ion_ is the ionomic age of the sample as determined by the ionomic marker, Age_chron_ is the chronological age of the same samples, and summation is performed over all *n* samples). The kidney clock was based on 12 elements (with most contributing being Na, Cu, and Zn), the pancreas clock on 15 elements (most contributions from Na, P, S, Zn, and Cd), and the testis clock on 16 elements (most contributing are Na, P, S, and K).

We were unable to construct a multi‐tissue ionomic clock accepting input from samples of all collected tissues (the error of such a multi‐tissue clock was dominated by the contributions of the tissues—muscle, lung, and especially liver). It was possible to construct a multi‐tissue clock accepting input only from kidney, pancreas, and testis samples, but the accuracy of the three‐tissue clock (mean absolute error in age identification for test samples ~4.2 months) was lower than that of the single tissue clocks. We attribute the low accuracy of this multi‐tissue clock to the fact that different elements contributed to the clocks developed for kidney, pancreas, and testis (with the exception of Na). For the same reason, we found that the application of clocks developed for a particular tissue failed when applied to other tissues (with the best mean absolute error of 3.8 months achieved when the kidney clock was applied to pancreas samples).

**Figure 6:**
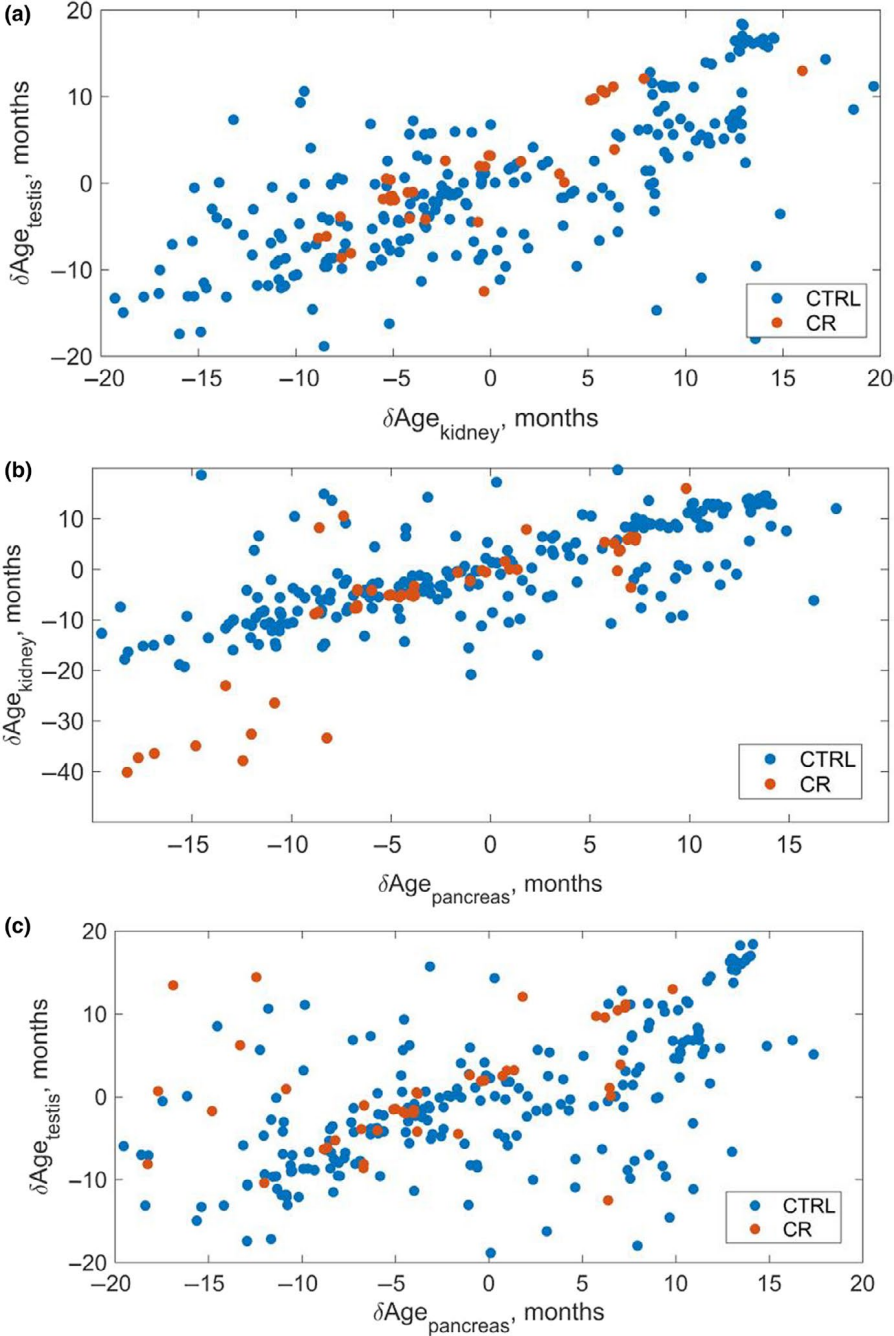
Displacement of ionomic age with respect to chronological age in different tissues. (a) Kidney versus testis. δAge is defined as a difference between the ionomic age of a sample as measured by a particular one‐tissue marker (kidney and testis correspondingly) and the chronological age of the sample. While the constructed ionomic markers of age are essentially different in different tissues (Figure 5), samples with higher displacement of the kidney ionomic age with respect to chronological age generally correspond to those with similarly higher displacement of the testis ionomic age with respect to the chronological age pointing toward biological significance of the measured ionomic age. Blue corresponds to mice on control diet, red—to mice on the calorie restriction (CR) diet. (b) The same for pancreas versus kidney. (c) The same for pancreas versus testis

In order to analyze outliers in terms of detected ionomic age, we compared $\delta \text{Age}={\text{Age}}_{\text{ion}}-{\text{Age}}_{\text{chron}}$ across different tissues and samples (Figure 6). It was found that mice with the observed higher displacement of biological (ionomic) age with respect to chronological age in one tissue were typically characterized by a similarly higher displacement of biological age with respect to the chronological one in another tissue. Given that ionomic markers of age developed here for different tissues have significantly different structures in different tissues with different elements contributing to the markers (Figure 5d,i,k), this points toward the biological significance of the ionomic age measured in different tissues by the developed markers.

### 2.7 Ionomic clocks report the effect of CR on the biological age of mice

The kidney (Figure 5b) and pancreas (Figure 5g) clocks revealed a steady increase in predicted age of mice on a standard diet as well as of CR mice (*p* = .01 for kidney and *p* = .03 for pancreas, one‐sided *t* test). The average difference between the chronological age of CR samples and their predicted biological age was ~8 months for both kidney and pancreas (to obtain this estimate, we calculated $\delta \text{Age}={\text{Age}}_{\text{chron}}-{\text{Age}}_{\text{ionomic}}$ for every sample and then averaged δAge across different samples). The testis ionomic clock did not discriminate between the mice on standard and CR diets (Figure 5i,j). It is possible that CR unequally affects different organs of mice, and the identification of organs that respond well to CR based on the ionomic clock provides an opportunity for broader application of these clocks to both address basic questions in the biology of aging and test longevity interventions.

## 3 DISCUSSION

In this work, we examined ionome remodeling across organs, ages, and diets, providing novel insights into the aging process and the biology of elements, and leading to the development of novel biomarkers of age. Similar to the previous cross‐species ionome analyses (Ma et al., 2015), our data indicate that the element patterns segregate based on organs and that the isotopes of the same element are utilized in a highly similar manner. In the current study, we also found that the ionomes of different tissues respond to diet. We observed many differences and common patterns in the distribution of elements across organs and found that the organ ionome is stable across the entire adult lifespan.

We observed that trace elements Mn, Cu, Zn, and Mo formed a cluster. Mo utilization, and specifically the biosynthesis of MoCo, is known to be dependent on Cu metabolism (Hänsch & Mendel, 2009). There are many other links among the elements of this cluster. For example, Zn is transported by ceruloplasmin, which is the most common cuproprotein in the blood. Both Zn and Cu are also used in the active site of the Cu–Zn superoxide dismutase, an abundant antioxidant enzyme. In addition, Zn toxicity can result in a lower Cu levels, whereas excessive intake of Zn prevents the utilization of Cu (Fraga, 2005; Council, 1989). Muscle and pancreas had higher Ca and Mg levels compared to other organs. Both elements possess important roles in muscle physiology, as Mg can stimulate the uptake of Ca ion during muscle contraction.

Previous ionome studies focused on the elemental composition across species and changes in elements in particular organs between young and old organisms of the same species (Takahashi et al., 2001). Instead, we sought to carry out a comprehensive analysis of element levels across different organs and diets as well as throughout the whole mouse lifespan. As such, we examined a large tissue collection of C57BL/6 mice representing 16 age groups, from 3 to 35 months old, and also included four age groups of mice (10–27 months old) subjected to CR.

The resulting dataset led to additional interesting insights. We observed that, in most tissues, the levels of most elements decreased with age. This is consistent with the acquired deficiency of elements as organisms age. However, some elements showed contrasting patterns in different tissues, and some increased with age, for example, Co in the brain. In addition, some elements displayed extreme changes in the oldest animals. In particular, many 34‐ and 35‐month old mice had extremely high levels of Ca in the brain and testis. This may be due to calcification of these organs in the oldest ages. It is known that abnormal calcification in the human brain is related to neurodegenerative diseases and tumorigenesis, and that testis calcification is also associated with cancer. Another interesting observation was that the levels of elements showed increased variability with age. This trend applied to most tissues analyzed, with the strongest effects in the kidney and brain. Increased variability in molecular analyses is a known characteristic of aging. It applies to gene expression, DNA methylation patterns, metabolite levels, and now to elemental composition of mouse tissues. At the same time, we are aware that our aged mice may be under the influence of a survivor bias due to the high mortality in the oldest age groups. Therefore, the trend we found may also be relevant to the adaptation to environment. Also, it was pointed out that the weight of mouse organs changes with age; for example, the weight of liver and kidney increases while that of testis decreases (Lessard‐Beaudoin, Laroche, Demers, Grenier, & Graham, 2015). Such changes may be a contributor to the age‐related changes.

Our work revealed stark differences in the levels of As and some other elements in CR mice. To figure out a possible source of this effect, we examined the dietary intake differences in trace elements between CR and standard diet mice. Interestingly, both vitamin B12 and Co contents of the CR diet were lower than that of control diet, pointing against the possibility that Co accumulated due to increased intake. One possibility is that the turnover of vitamin B12 is slower in CR mice due to the limited energy intake and redistribution of nutrient pathways, causing the accumulation of this element in the body. A similar case may be related to the increased levels of As in CR mice. Metabolism of As in mice is not fully understood, although it is known that arsenic methyltransferase is a key enzyme that helps remove this element from tissues. We examined the gene expression data of CR mice and found a significant upregulation of arsenic methyltransferase by CR in the liver (Tyshkovskiy et al., 2019). We hypothesize that the increased expression of this enzyme is a response to the increased levels of As in CR animals.

Analyses of CR across ages and tissues offered additional insights. At the level of the organismal ionome, we observed a steady transition of control animals from young to old ages. The CR trajectory moved in the same direction, but was shifted. This suggests that CR remodels metabolism of the organism such that the animals age through a different trajectory. In other words, rather than making a CR animal more resembling a younger animal, CR makes it different. However, at the level of individual tissues, most notably the kidney and muscle, CR samples were closer to the younger control samples.

Assessing the rate of aging is crucial in aging research, as age‐related phenotypes cannot tell the effect of longevity interventions precisely. Studies on longevity interventions traditionally relied on mortality assays, which is time‐consuming and expensive. From this perspective, a reliable biomarker of age is necessary for understanding the systematic changes associated with aging and testing longevity interventions. Many aging biomarkers have been previously discovered, including the most robust biomarker known as the DNA methylation clock (Horvath, 2013; Meer, Podolskiy, Tyshkovskiy, & Gladyshev, 2018; Petkovich et al., 2017). It is important to note that newly developed clocks based on pyrosequencing can assess the biological age based on only a handful of sites (Weidner et al., 2014). However, the substantial cost of methylation clocks restricts its use on a massive scale. We found that while the organismal ionome as a whole did not show a sufficient predictive power, the individual ionomes of some organs were highly predictive. In particular, analyses of kidney, pancreas, and testes led to the development of robust aging biomarkers, the ionomic clocks, in these tissues. Moreover, the kidney and pancreas clocks reported the effect of CR; that is, CR mice were younger than control animals of the same chronological age based on these clocks. It would be interesting to more broadly apply these clocks in the future to examine additional longevity interventions. It is important to note that methylation clock requires significant postharvest sample processing and sequencing, while in comparison the ionome clock requires only nitric acid digestion after sample harvesting and is subject to a much lower cost. The ease of application of ionomic clocks may make them viable candidates for further analyses in the biology of aging and for tests of longevity interventions.

## 4 EXPERIMENTAL PROCEDURES

### 4.1 Tissue samples

Mice (C57BL/6 males) used in the study were obtained from the NIA Aged Rodent Colony. The animals were raised in the Charles River barrier facility with wood shaving bedding, 12 hr/12 hr light/dark cycle, and free access to pH 7.0–7.5 water with 4–6 ppm chlorine. These mice were given sterilized NIH31 or NIH31‐fortified (CR animals only) diets. They were transported to the animal facility at Brigham and Women's Hospital and sacrificed, and their tissues were immediately collected and stored at −80°C until use. The mice represented 16 age groups, from 3 to 35 months old. A separate group of mice was subjected to CR in the same facility. Calorie restriction started at 14 weeks of age and continued until the four time points at which the animals were sacrificed, and their tissues were collected as was done for control mice. The experimental protocols were approved by the local Institutional Animal Care and Use Committee. Further information about the samples can be found in Table S1.

### 4.2 Quantification of elements

Inductively coupled plasma‐mass spectrometry was applied to characterize element levels in the brain, lung, heart, testis, liver, muscle, pancreas, and kidney of mice as described previously (Ma et al., 2015). Tissues were originally cut and examined in the range of 10–90 mg wet weight, and following method development, approximately 10 mg of tissue was used for each measurement. The mice were males, and we had two technical replicates (tissues from same animals with repeat runs in different batches) and four to five biological replicates (tissues from different animals with the same age and diet; Table S1). Sample digestion and subsequent analyses were performed at the University of Nebraska–Lincoln spectroscopy facility equipped with Agilent Technologies ICP‐MS 7500cx series (Agilent Technologies) and SC autosampler (Elemental Scientific). Samples from tissues other than the brain were digested overnight in three times weight of 70% nitric acid at 70°C, while brain samples required a secondary digestion for 3–4 hr at 70°C with 15% hydrogen peroxide. Thirty isotopes from 20 elements were originally analyzed. The levels of Li (μg), B (ng), Na (mg), Mg (μg), P (mg), S (mg), K (mg), Ca (μg), V(μg), Cr(μg), Mn (ng), Fe (μg), Co (ng), Ni (ng), Cu (μg), Zn (μg), As (ng), Se (ng), Mo (ng), and Cd (ng), per gram of tissue digested in nitric acid, were quantified with spike‐in 50 μg/L Ga as internal standard, using the sample preparation and data collection method described previously (Ma et al., 2015). Two isotopes were measured, respectively, for Ca (^40^Ca, ^44^Ca), V (^50^V, ^51^V), Cr (^52^Cr, ^53^Cr), Fe (^56^Fe, ^57^Fe), Ni (^60^Ni, ^62^Ni), Cu (^63^Cu, ^65^Cu), Zn (^64^Zn, ^66^Zn), Se (^78^Se, ^82^Se), Mo (^95^Mo, ^98^Mo), and Cd (^111^Cd, ^112^Cd). Each organ was subjected to an independent ICP‐MS run, running on two separate plates. The analysis used a collision cell filled with 3.5 ml/min of H2 and 1.5 ml/min of He with an Ar carrier flow of 0.9 L/min and Ar make‐up flow of 0.15 L/min, and RF power of 1,500 W.

### 4.3 Data processing and filtering

The data with lower element abundance value than the characteristic equipment noise (determined by the parameter limits of quantification [LOQ] estimated according to the equation LOQ = 3.33*limits of detection [DLs] + background equivalent concentration [BEC]) were omitted. Samples for which the observed element abundance values were below mean blank concentration were also removed from the analysis. Thirty isotopes representing 20 elements for which the number of excluded samples according to this criterion exceeded 50% were then removed from the analysis. The resulting data included 651 distinct tissue samples, which were then analyzed for batch effect. We had 1–2 technical replicates per tissue sample, resulting in 1,271 samples in total. The considered batch effect factors included location on the periphery of the plate, position of lanes on the plate, and plate number. Principal component analysis revealed that the main contributor to the batch effect was the difference between distinct plates (Figure S1). We thus processed the data to remove batch effect due to the plate number: For each tissue separately, we fit element abundances (response variable) and plate number (explanatory variable) in a linear model and considered the residuals as corrected element abundances (Figure S2). Namely, the data matrix *X* was fit to a linear model $X=\alpha_{0}+\alpha_{1}\cdot \text{Age}+\alpha_{2}\cdot N+\epsilon$, where *N* is the plate number. After coefficients of the multiple linear regression were determined, the term *α*_2_·*N* was subtracted, with the resulting expression *X* − *α*_2_·*N* subjected to further analysis. Finally, the processed data were standardized by calculating the row average, subtracting it from the data and dividing the result by the standard deviation within the row. This scaled data, with an average of 0 and standard deviation of 1, were then used for comparison among organs and between control and CR samples. For PCA analysis, we applied R function prcomp() on the data used. For the analysis by organs, we used the data from control tissue samples (1,016 measurements from 524 samples). For the analysis across ages and diets, we used the data from all control and CR tissue samples (1,271 measurements from 651 samples). For correction of *p*‐values in multiple testing, we used Benjamini–Hochberg procedure to adjust our *p*‐values between control and CR group.

### 4.4 Establishing the ionome‐based biomarkers of aging in mice

We developed several biological markers of age using elastic net regression of filtered ionome data to chronological age of samples using previously described procedures (Petkovich et al., 2017). The different markers which we developed were based on the ionomes characterizing eight mouse organs (brain, lung, heart, testis, liver, muscle, pancreas, and kidney). To increase accuracy of biomarkers of age (by increasing the number of samples per age category), we combined samples into eight age categories separated by the period of 5 months. The dataset used to train the markers included samples collected from 128 mice in standard diet and 32 mice subjected to calorie restricted diet, with two biological replicates in each group. Each constructed elastic net regression‐based biological marker of age was defined as a vector of weights *w* of abundances *x_n_* of different elements in the linear combination $X\left(t\right)=w_{0}+w_{1}x_{1}\left(t\right)+w_{2}x_{2}\left(t\right)+\;\cdots \;+w_{n}x_{n}\left(t\right)$ (here *t* is the age of a sample) minimizing the target function $F\left(t\right)={\left|X-t\right|}_{2}+\frac{\lambda }{2}\left(\frac{1}{2}\left(w\cdot w\right)+{\left|w\right|}_{1}\right)$. For every marker being constructed, scanning for different values of Tikhonov regularization parameter *λ* was performed with 20‐fold cross‐validation (representing a random separation of the original dataset into 20 subsets, 19 of which being used to train the marker and the remaining one—to test it). The cross‐validation deviance was estimated for every value in the scanned range of Tikhonov regularization parameters *λ*, and the value $\lambda_{*}$corresponding to the minimum of the cross‐validation deviance was identified. The elastic net regression‐based markers were constructed in MATLAB using lassoglm procedure.

## CONFLICTS OF INTEREST

None declared.

## AUTHOR CONTRIBUTIONS

V.N.G. coordinated the study. B.Z., D.I.P., and M.M. carried out data analyses. B.Z. prepared samples for ICP‐MS. J.S. carried out ICP‐MS measurements. All authors contributed to data interpretation. B.Z., D.I.P., and V.N.G. wrote the paper with input from all authors.

## Data Availability

The data that supports the findings of this study are available in the Supporting Information of this article.
