# Supplementary material for: Systematic age‐, organ‐, and diet‐associated ionome remodeling and the development of ionomic aging clocks
Source: Aging Cell. 2020 Apr 23;19(5):e13119. doi: 10.1111/acel.13119 (PMC7253066; doi:10.1111/acel.13119)
Supplement: Supplementary file 1 [file ACEL-19-e13119-s001.pdf]

Supporting Information

Fig. S1. Principal Component Analysis showing factors contributing to batch effect.
Heatmap shows the contribution of age, plate, lane and lane position to the first two principal components in different organs.

Fig. S2. Batch effect removal by linear model.
(A) ANOVA test on age, batch and diet before batch effect correction. Bar plot shows magnitude of p-value on elements. **(B)** ANOVA test on age, batch and diet after batch effect correction by linear model. Bar plot shows magnitude of p-value on elements.

Fig. S3. Heatmap overview of element distribution in various organs.
Each row represents an element or isotope. Each column represents a particular biological sample. Elements with the level lower than noise in certain tissues are shown in grey. Clustering was performed using complete-linkage method with Euclidean distance measure.

Fig. S4. Overview of element changes with age.
Dot plots show changes of element composition with age. Each plot represents changes in the indicated element with age in the indicated organ. The X axis represents the age of the animal in month and the y axis represents the original composition of elements for Na (mg/g), Mg (μg/g), P (mg/g), S (mg/g), K (mg/g), Ca (μg/g) , Mn (ng/g), Fe (μg/g), Co (ng/g), Cu (μg/g), Zn (μg/g), Se (ng/g), and Mo (ng/g).

Fig. S5. Principal Component Analysis of samples from 6 organs.
Principal Component Analysis of samples from brain, lung, heart, testis, liver and pancreas based on the age and diet. Percentage variation explained by the first 3 components is shown.

Fig. S6. Changes of element levels in CR mice with age.
(A) Spearman correlation coefficient between age and element levels in CR (calorie restriction) mice. **(B)** Spearman correlation coefficient between age and element coefficient of variation in CR mice. **(C)** Boxplot of (1) Residue applied to control (Normal) and CR group on Age to PC1 linear regression of control group from **Fig. 3E** (upper left); (1) Control and CR group in PC2 from **Fig. 3E** (upper right); (1) Residue applied to control and CR group on Age to PC2 linear regression of Muscle control group from **Fig. 4A** (lower left); (1) Residue applied to control and CR group on Age to PC2 linear regression of Kidney control group from **Fig. 4A** (lower right); p value from Wilcoxon test scores are shown.

Table S1. General information on samples, including age, sex, diet and tissue types.

Table S2. Content of elements in each tissue sample.

Table S3. Principal components. This table features the loading values in Principal Component Analysis in Figure 3E.

Table S4. Element content of diets for control and CR groups.

Fig. S1

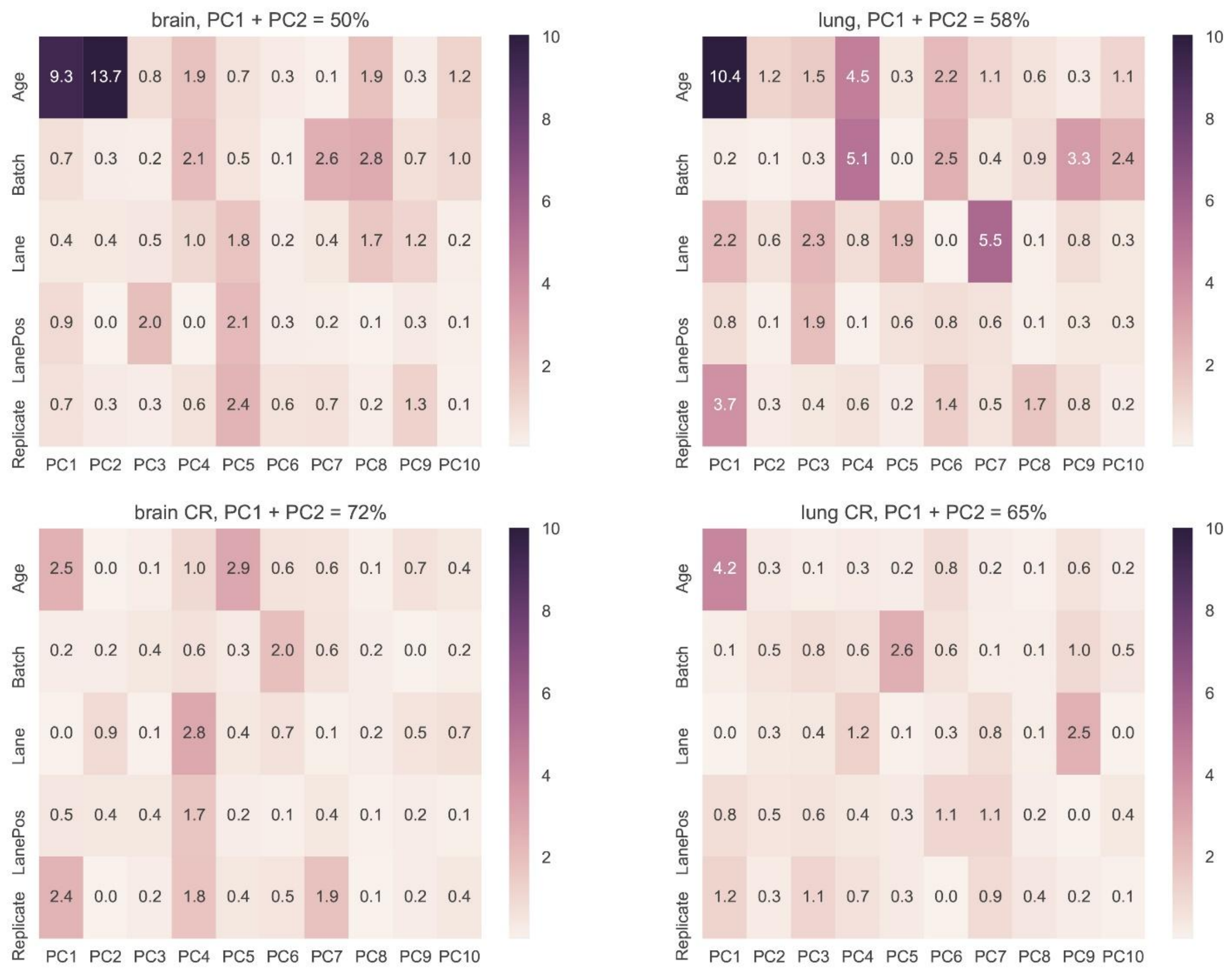
brain, PC1 + PC2 = 50%
lung, PC1 + PC2 = 58%
brain CR, PC1 + PC2 = 72%
lung CR, PC1 + PC2 = 65%
Age
Batch
Lane
LanePos
Replicate
PC1 PC2 PC3 PC4 PC5 PC6 PC7 PC8 PC9 PC10
9.3 13.7 0.8 1.9 0.7 0.3 0.1 1.9 0.3 1.2
0.7 0.3 0.2 2.1 0.5 0.1 2.6 2.8 0.7 1.0
0.4 0.4 0.5 1.0 1.8 0.2 0.4 1.7 1.2 0.2
0.9 0.0 2.0 0.0 2.1 0.3 0.2 0.1 0.3 0.1
0.7 0.3 0.3 0.6 2.4 0.6 0.7 0.2 1.3 0.1
10.4 1.2 1.5 4.5 0.3 2.2 1.1 0.6 0.3 1.1
0.2 0.1 0.3 5.1 0.0 2.5 0.4 0.9 3.3 2.4
2.2 0.6 2.3 0.8 1.9 0.0 5.5 0.1 0.8 0.3
0.8 0.1 1.9 0.1 0.6 0.8 0.6 0.1 0.3 0.3
3.7 0.3 0.4 0.6 0.2 1.4 0.5 1.7 0.8 0.2
2.5 0.0 0.1 1.0 2.9 0.6 0.6 0.1 0.7 0.4
0.2 0.2 0.4 0.6 0.3 2.0 0.6 0.2 0.0 0.2
0.0 0.9 0.1 2.8 0.4 0.7 0.1 0.2 0.5 0.7
0.5 0.4 0.4 1.7 0.2 0.1 0.4 0.1 0.2 0.1
2.4 0.0 0.2 1.8 0.4 0.5 1.9 0.1 0.2 0.4
4.2 0.3 0.1 0.3 0.2 0.8 0.2 0.1 0.6 0.2
0.1 0.5 0.8 0.6 2.6 0.6 0.1 0.1 1.0 0.5
0.0 0.3 0.4 1.2 0.1 0.3 0.8 0.1 2.5 0.0
0.8 0.5 0.6 0.4 0.3 1.1 1.1 0.2 0.0 0.4
1.2 0.3 1.1 0.7 0.3 0.0 0.9 0.4 0.2 0.1
10
8
6
4
2

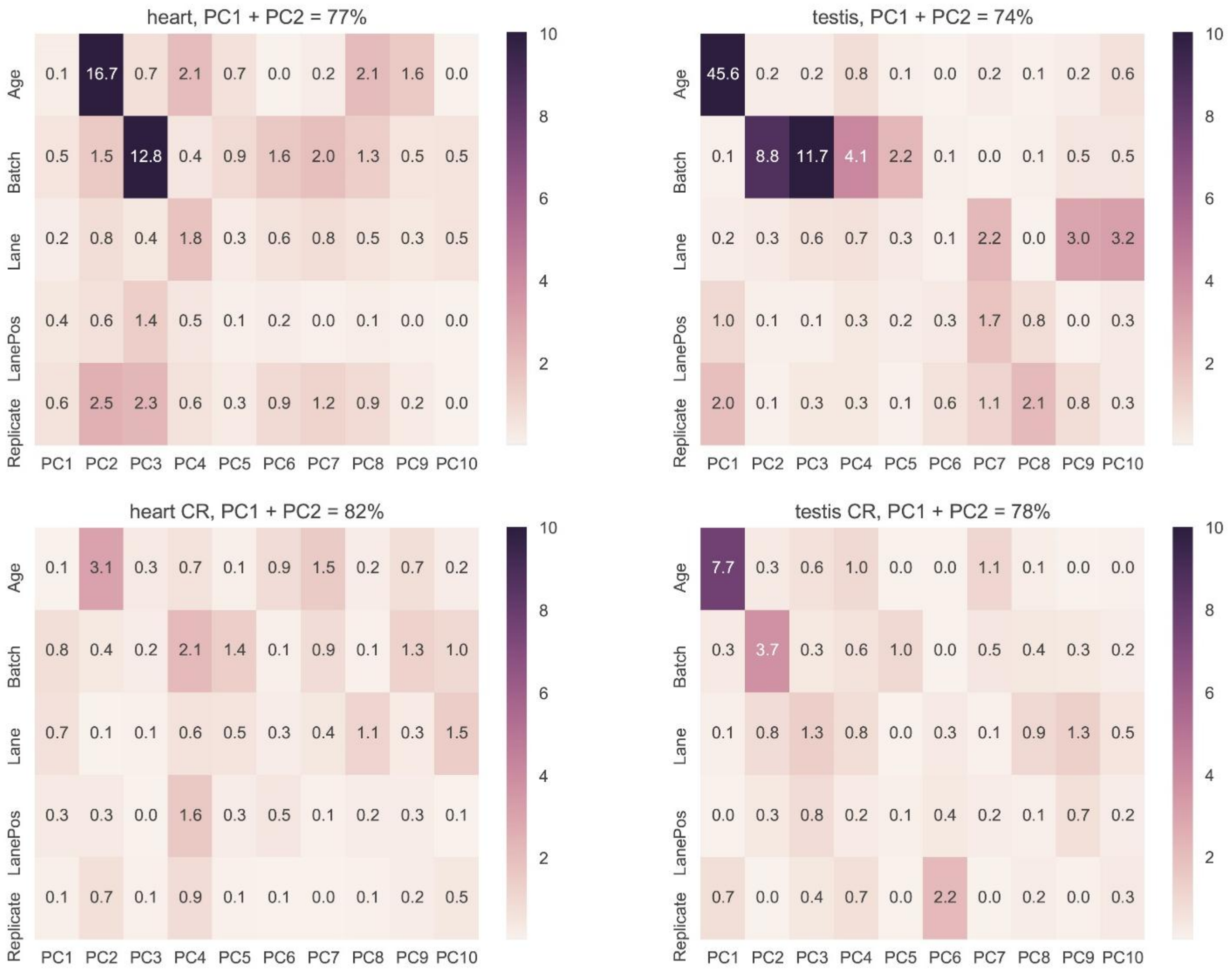
heart, PC1 + PC2 = 77%
Age 0.1 16.7 0.7 2.1 0.7 0.0 0.2 2.1 1.6 0.0
Batch 0.5 1.5 12.8 0.4 0.9 1.6 2.0 1.3 0.5 0.5
Lane 0.2 0.8 0.4 1.8 0.3 0.6 0.8 0.5 0.3 0.5
LanePos 0.4 0.6 1.4 0.5 0.1 0.2 0.0 0.1 0.0 0.0
Replicate 0.6 2.5 2.3 0.6 0.3 0.9 1.2 0.9 0.2 0.0
PC1 PC2 PC3 PC4 PC5 PC6 PC7 PC8 PC9 PC10
10 8 6 4 2
testis, PC1 + PC2 = 74%
Age 45.6 0.2 0.2 0.8 0.1 0.0 0.2 0.1 0.2 0.6
Batch 0.1 8.8 11.7 4.1 2.2 0.1 0.0 0.1 0.5 0.5
Lane 0.2 0.3 0.6 0.7 0.3 0.1 2.2 0.0 3.0 3.2
LanePos 1.0 0.1 0.1 0.3 0.2 0.3 1.7 0.8 0.0 0.3
Replicate 2.0 0.1 0.3 0.3 0.1 0.6 1.1 2.1 0.8 0.3
PC1 PC2 PC3 PC4 PC5 PC6 PC7 PC8 PC9 PC10
10 8 6 4 2
heart CR, PC1 + PC2 = 82%
Age 0.1 3.1 0.3 0.7 0.1 0.9 1.5 0.2 0.7 0.2
Batch 0.8 0.4 0.2 2.1 1.4 0.1 0.9 0.1 1.3 1.0
Lane 0.7 0.1 0.1 0.6 0.5 0.3 0.4 1.1 0.3 1.5
LanePos 0.3 0.3 0.0 1.6 0.3 0.5 0.1 0.2 0.3 0.1
Replicate 0.1 0.7 0.1 0.9 0.1 0.1 0.0 0.1 0.2 0.5
PC1 PC2 PC3 PC4 PC5 PC6 PC7 PC8 PC9 PC10
10 8 6 4 2
testis CR, PC1 + PC2 = 78%
Age 7.7 0.3 0.6 1.0 0.0 0.0 1.1 0.1 0.0 0.0
Batch 0.3 3.7 0.3 0.6 1.0 0.0 0.5 0.4 0.3 0.2
Lane 0.1 0.8 1.3 0.8 0.0 0.3 0.1 0.9 1.3 0.5
LanePos 0.0 0.3 0.8 0.2 0.1 0.4 0.2 0.1 0.7 0.2
Replicate 0.7 0.0 0.4 0.7 0.0 2.2 0.0 0.2 0.0 0.3
PC1 PC2 PC3 PC4 PC5 PC6 PC7 PC8 PC9 PC10
10 8 6 4 2

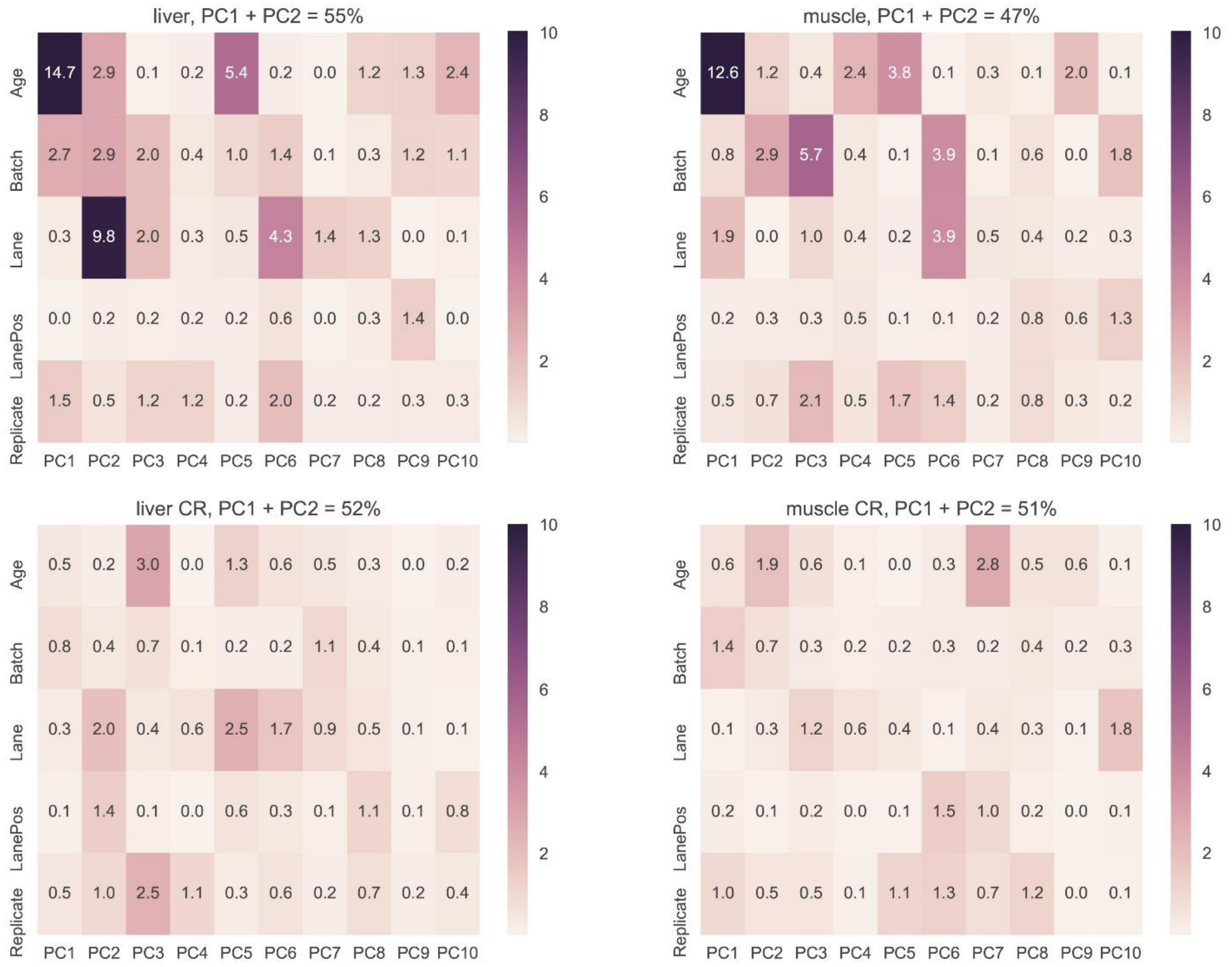
liver, PC1 + PC2 = 55%
Age 14.7 2.9 0.1 0.2 5.4 0.2 0.0 1.2 1.3 2.4
Batch 2.7 2.9 2.0 0.4 1.0 1.4 0.1 0.3 1.2 1.1
Lane 0.3 9.8 2.0 0.3 0.5 4.3 1.4 1.3 0.0 0.1
LanePos 0.0 0.2 0.2 0.2 0.2 0.6 0.0 0.3 1.4 0.0
Replicate 1.5 0.5 1.2 1.2 0.2 2.0 0.2 0.2 0.3 0.3
PC1 PC2 PC3 PC4 PC5 PC6 PC7 PC8 PC9 PC10
10 8 6 4 2
muscle, PC1 + PC2 = 47%
Age 12.6 1.2 0.4 2.4 3.8 0.1 0.3 0.1 2.0 0.1
Batch 0.8 2.9 5.7 0.4 0.1 3.9 0.1 0.6 0.0 1.8
Lane 1.9 0.0 1.0 0.4 0.2 3.9 0.5 0.4 0.2 0.3
LanePos 0.2 0.3 0.3 0.5 0.1 0.1 0.2 0.8 0.6 1.3
Replicate 0.5 0.7 2.1 0.5 1.7 1.4 0.2 0.8 0.3 0.2
PC1 PC2 PC3 PC4 PC5 PC6 PC7 PC8 PC9 PC10
10 8 6 4 2
liver CR, PC1 + PC2 = 52%
Age 0.5 0.2 3.0 0.0 1.3 0.6 0.5 0.3 0.0 0.2
Batch 0.8 0.4 0.7 0.1 0.2 0.2 1.1 0.4 0.1 0.1
Lane 0.3 2.0 0.4 0.6 2.5 1.7 0.9 0.5 0.1 0.1
LanePos 0.1 1.4 0.1 0.0 0.6 0.3 0.1 1.1 0.1 0.8
Replicate 0.5 1.0 2.5 1.1 0.3 0.6 0.2 0.7 0.2 0.4
PC1 PC2 PC3 PC4 PC5 PC6 PC7 PC8 PC9 PC10
10 8 6 4 2
muscle CR, PC1 + PC2 = 51%
Age 0.6 1.9 0.6 0.1 0.0 0.3 2.8 0.5 0.6 0.1
Batch 1.4 0.7 0.3 0.2 0.2 0.3 0.2 0.4 0.2 0.3
Lane 0.1 0.3 1.2 0.6 0.4 0.1 0.4 0.3 0.1 1.8
LanePos 0.2 0.1 0.2 0.0 0.1 1.5 1.0 0.2 0.0 0.1
Replicate 1.0 0.5 0.5 0.1 1.1 1.3 0.7 1.2 0.0 0.1
PC1 PC2 PC3 PC4 PC5 PC6 PC7 PC8 PC9 PC10
10 8 6 4 2

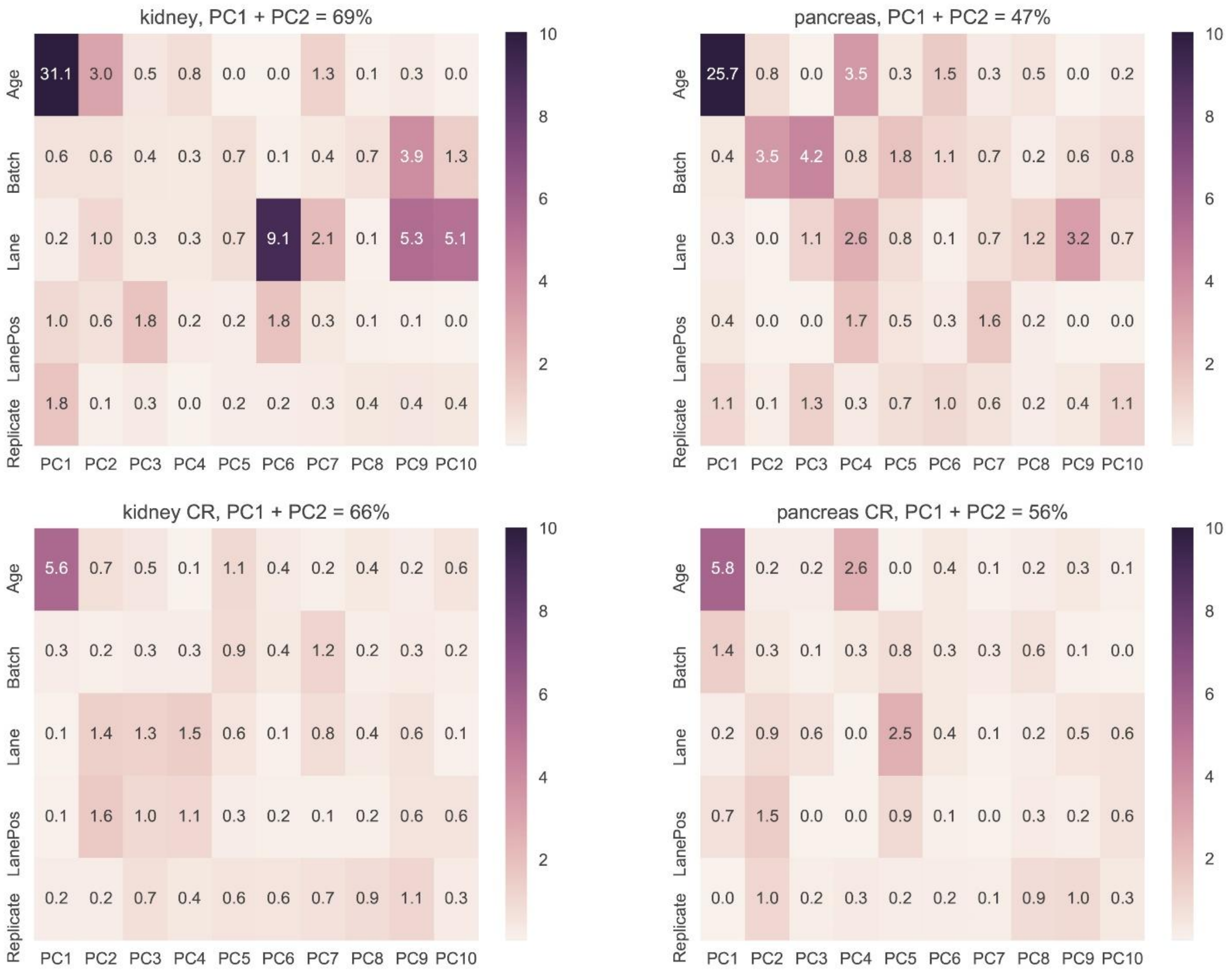
kidney, PC1 + PC2 = 69%
Age 31.1 3.0 0.5 0.8 0.0 0.0 1.3 0.1 0.3 0.0
Batch 0.6 0.6 0.4 0.3 0.7 0.1 0.4 0.7 3.9 1.3
Lane 0.2 1.0 0.3 0.3 0.7 9.1 2.1 0.1 5.3 5.1
LanePos 1.0 0.6 1.8 0.2 0.2 1.8 0.3 0.1 0.1 0.0
Replicate 1.8 0.1 0.3 0.0 0.2 0.2 0.3 0.4 0.4 0.4
PC1 PC2 PC3 PC4 PC5 PC6 PC7 PC8 PC9 PC10
10 8 6 4 2
pancreas, PC1 + PC2 = 47%
Age 25.7 0.8 0.0 3.5 0.3 1.5 0.3 0.5 0.0 0.2
Batch 0.4 3.5 4.2 0.8 1.8 1.1 0.7 0.2 0.6 0.8
Lane 0.3 0.0 1.1 2.6 0.8 0.1 0.7 1.2 3.2 0.7
LanePos 0.4 0.0 0.0 1.7 0.5 0.3 1.6 0.2 0.0 0.0
Replicate 1.1 0.1 1.3 0.3 0.7 1.0 0.6 0.2 0.4 1.1
PC1 PC2 PC3 PC4 PC5 PC6 PC7 PC8 PC9 PC10
10 8 6 4 2
kidney CR, PC1 + PC2 = 66%
Age 5.6 0.7 0.5 0.1 1.1 0.4 0.2 0.4 0.2 0.6
Batch 0.3 0.2 0.3 0.3 0.9 0.4 1.2 0.2 0.3 0.2
Lane 0.1 1.4 1.3 1.5 0.6 0.1 0.8 0.4 0.6 0.1
LanePos 0.1 1.6 1.0 1.1 0.3 0.2 0.1 0.2 0.6 0.6
Replicate 0.2 0.2 0.7 0.4 0.6 0.6 0.7 0.9 1.1 0.3
PC1 PC2 PC3 PC4 PC5 PC6 PC7 PC8 PC9 PC10
10 8 6 4 2
pancreas CR, PC1 + PC2 = 56%
Age 5.8 0.2 0.2 2.6 0.0 0.4 0.1 0.2 0.3 0.1
Batch 1.4 0.3 0.1 0.3 0.8 0.3 0.3 0.6 0.1 0.0
Lane 0.2 0.9 0.6 0.0 2.5 0.4 0.1 0.2 0.5 0.6
LanePos 0.7 1.5 0.0 0.0 0.9 0.1 0.0 0.3 0.2 0.6
Replicate 0.0 1.0 0.2 0.3 0.2 0.2 0.1 0.9 1.0 0.3
PC1 PC2 PC3 PC4 PC5 PC6 PC7 PC8 PC9 PC10
10 8 6 4 2

Fig. S2
A

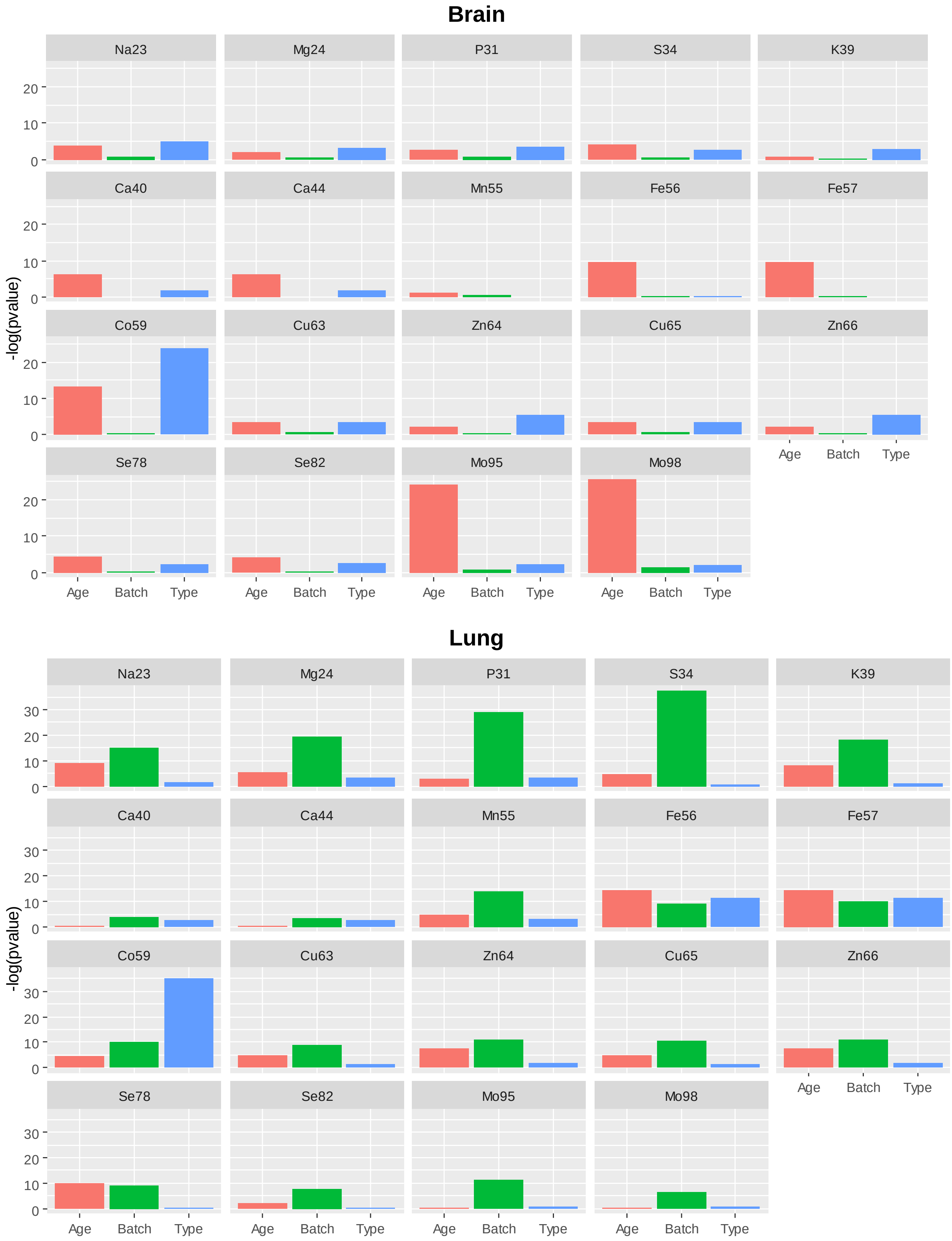
Brain
Na23
Mg24
P31
S34
K39
Ca40
Ca44
Mn55
Fe56
Fe57
Co59
Cu63
Zn64
Cu65
Zn66
Se78
Se82
Mo95
Mo98
-log(pvalue)
0
10
20
Age
Batch
Type
Lung
Na23
Mg24
P31
S34
K39
Ca40
Ca44
Mn55
Fe56
Fe57
Co59
Cu63
Zn64
Cu65
Zn66
Se78
Se82
Mo95
Mo98
-log(pvalue)
0
10
20
30
Age
Batch
Type

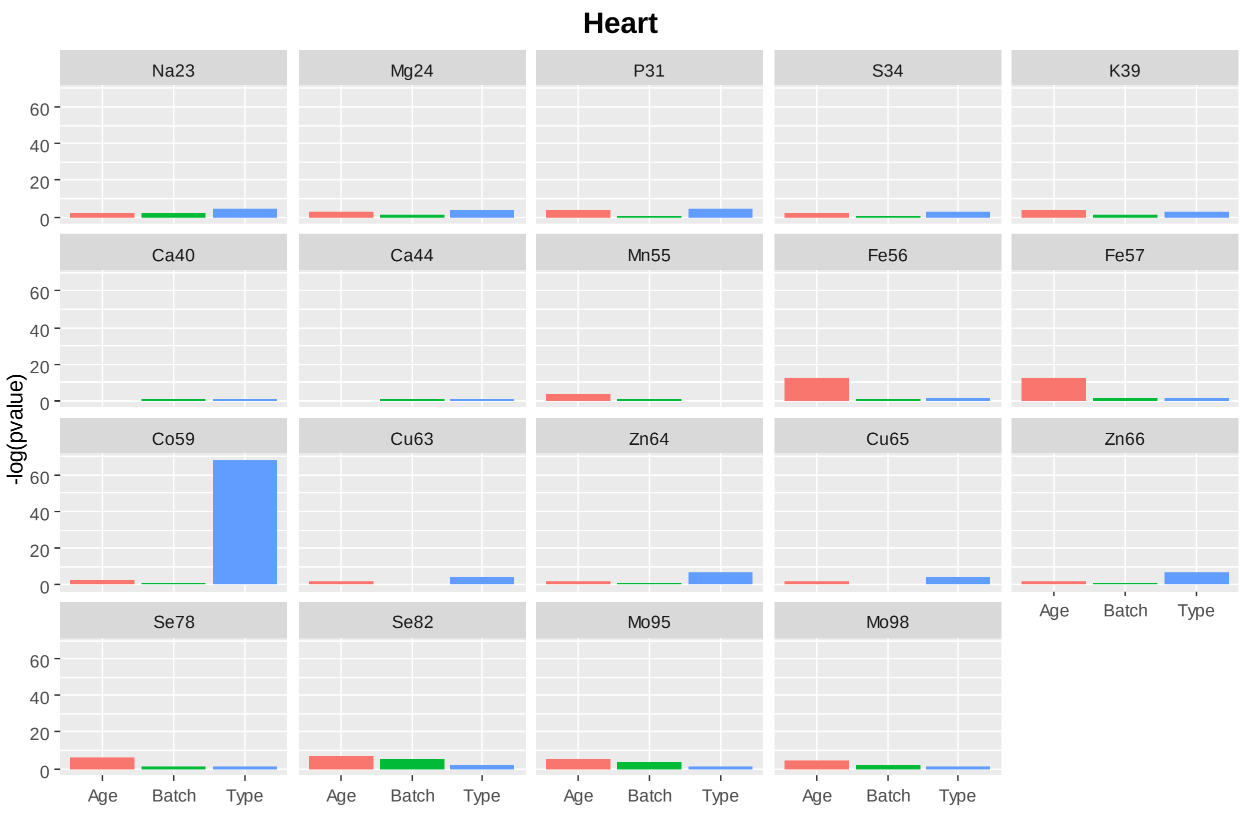
Heart
Na23
Mg24
P31
S34
K39
Ca40
Ca44
Mn55
Fe56
Fe57
Co59
Cu63
Zn64
Cu65
Zn66
Se78
Se82
Mo95
Mo98
-log(pvalue)
0
20
40
60
Age
Batch
Type

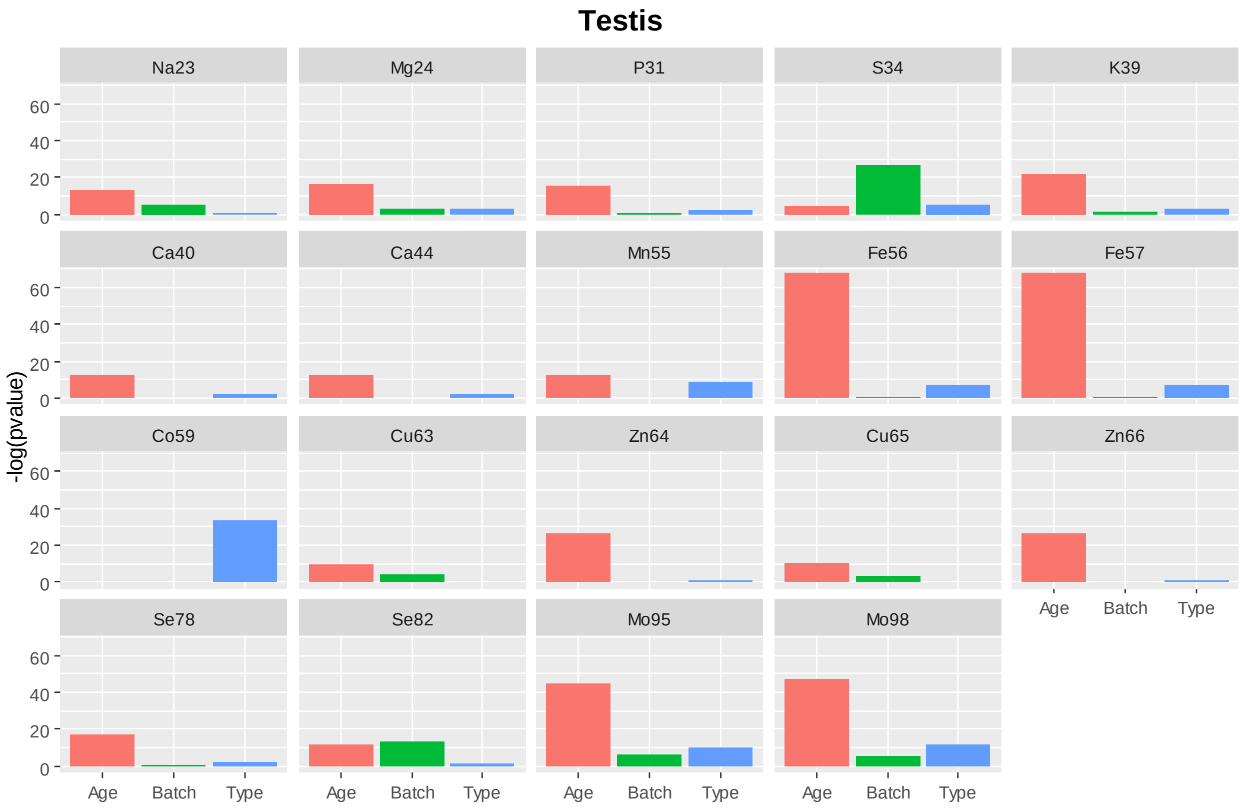
Testis
Na23
Mg24
P31
S34
K39
Ca40
Ca44
Mn55
Fe56
Fe57
Co59
Cu63
Zn64
Cu65
Zn66
Se78
Se82
Mo95
Mo98
-log(pvalue)
0
20
40
60
Age
Batch
Type

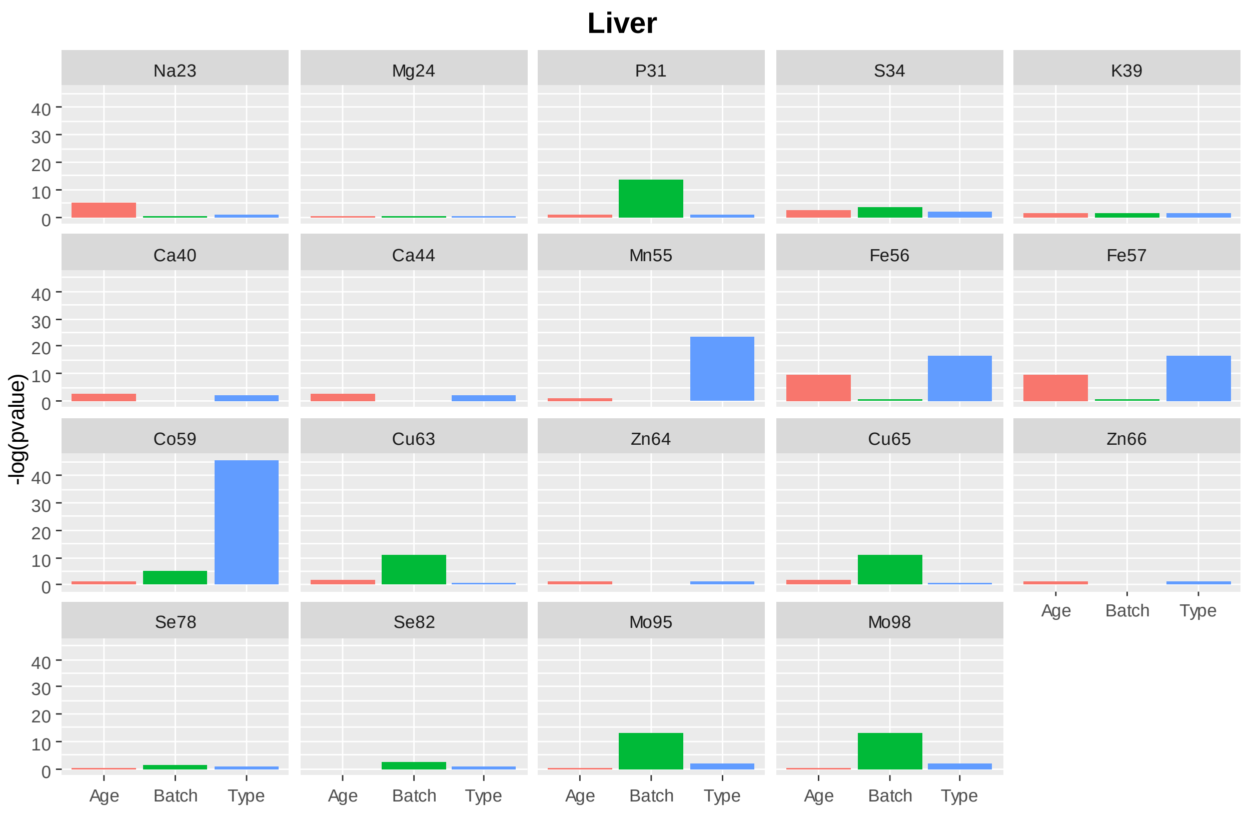
Liver
Na23
Mg24
P31
S34
K39
Ca40
Ca44
Mn55
Fe56
Fe57
Co59
Cu63
Zn64
Cu65
Zn66
Se78
Se82
Mo95
Mo98
-log(pvalue)
0
10
20
30
40
Age
Batch
Type

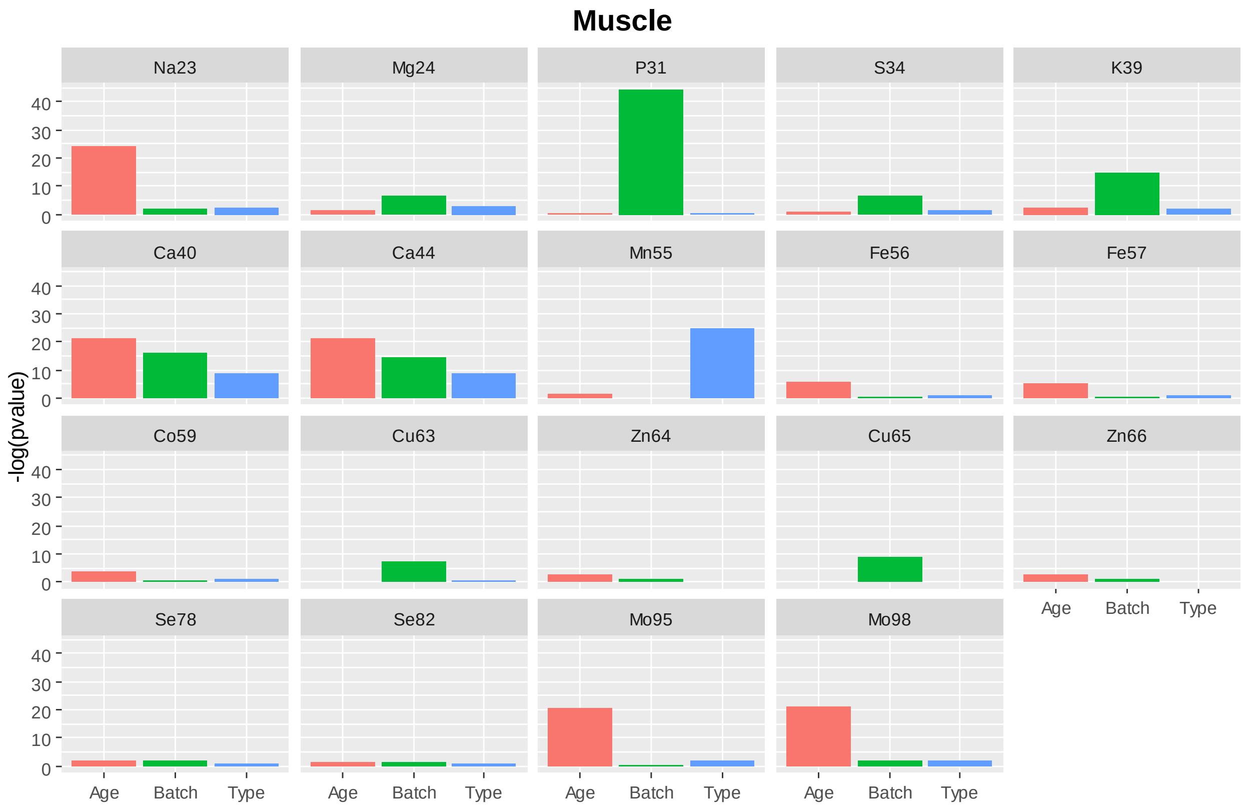
Muscle
Na23
Mg24
P31
S34
K39
Ca40
Ca44
Mn55
Fe56
Fe57
Co59
Cu63
Zn64
Cu65
Zn66
Se78
Se82
Mo95
Mo98
-log(pvalue)
0
10
20
30
40
Age
Batch
Type

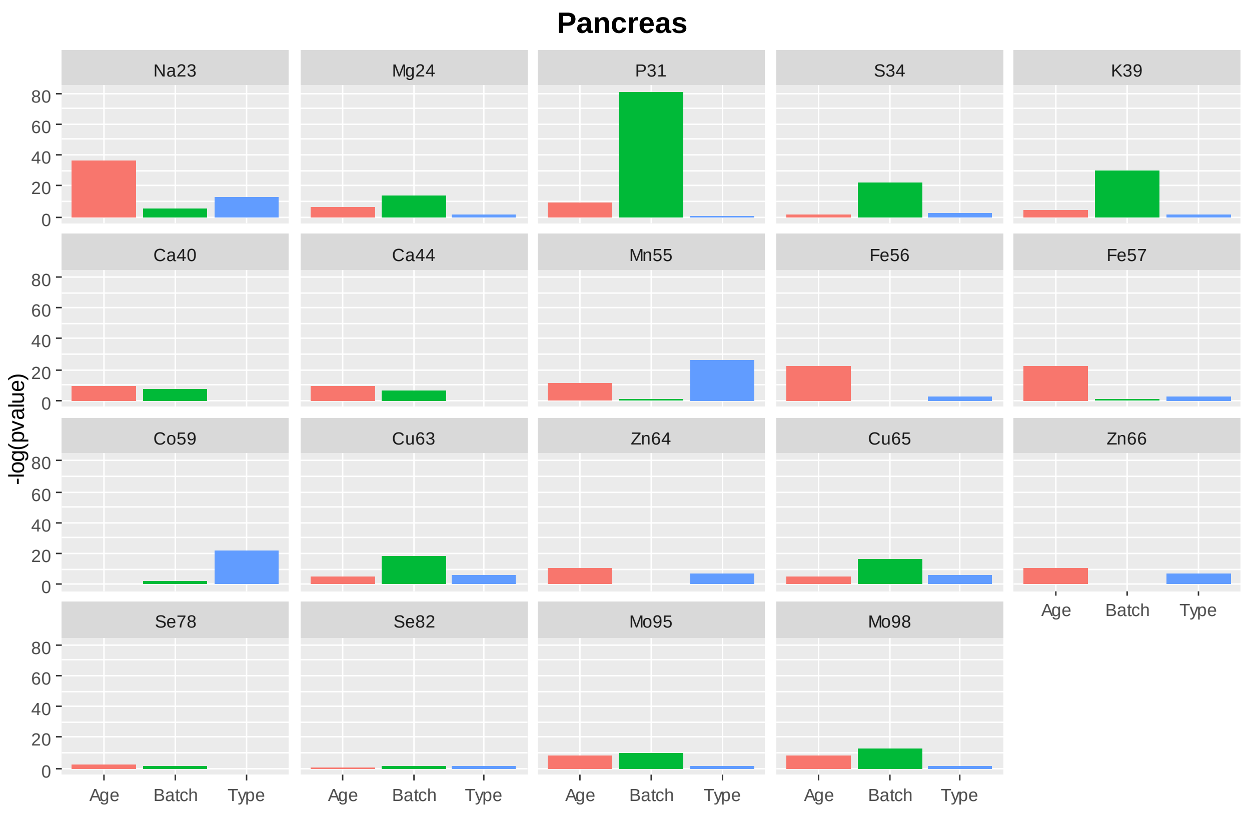
Pancreas
Na23
Mg24
P31
S34
K39
Ca40
Ca44
Mn55
Fe56
Fe57
Co59
Cu63
Zn64
Cu65
Zn66
Se78
Se82
Mo95
Mo98
-log(pvalue)
80
60
40
20
0
Age
Batch
Type

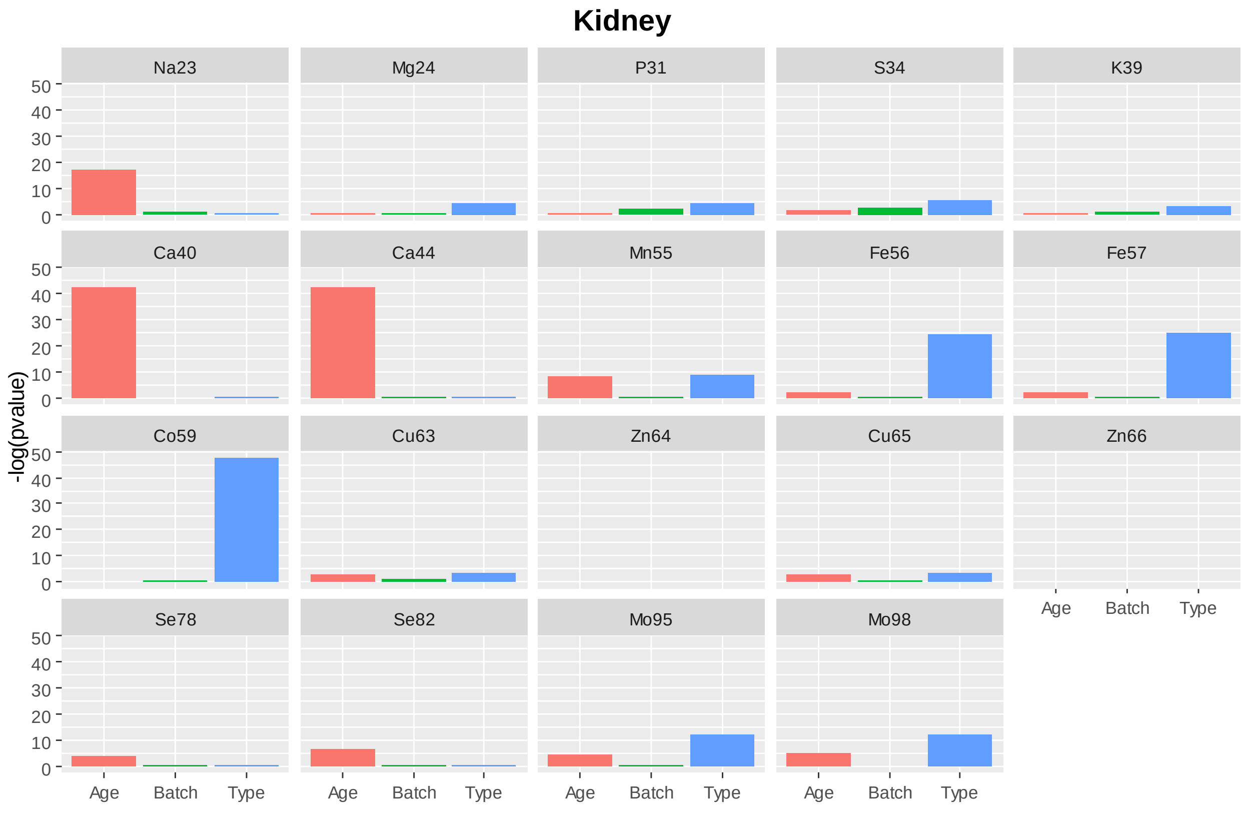
Kidney
Na23
Mg24
P31
S34
K39
Ca40
Ca44
Mn55
Fe56
Fe57
Co59
Cu63
Zn64
Cu65
Zn66
Se78
Se82
Mo95
Mo98
-log(pvalue)
50
40
30
20
10
0
Age
Batch
Type

B

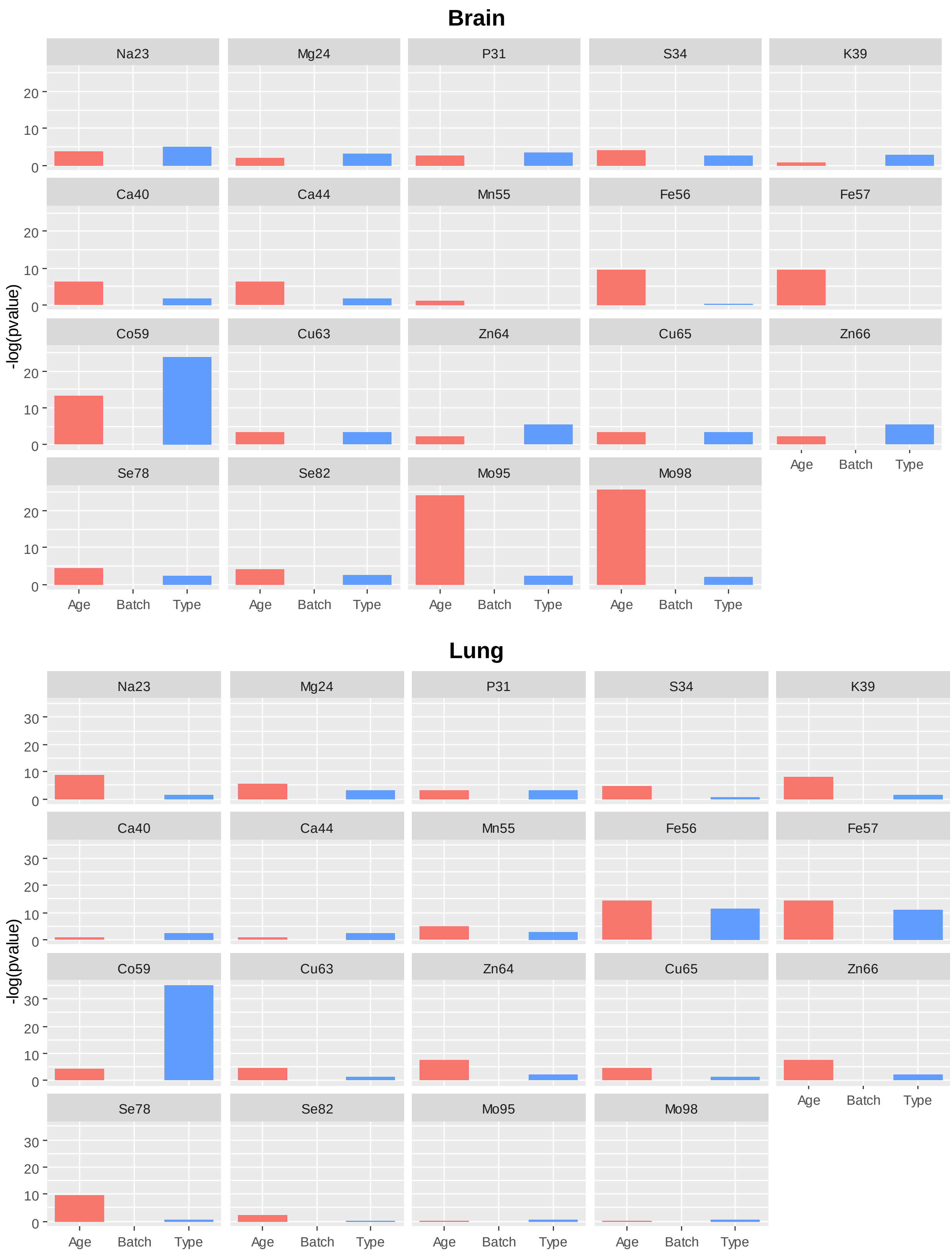
Brain
Na23
Mg24
P31
S34
K39
Ca40
Ca44
Mn55
Fe56
Fe57
Co59
Cu63
Zn64
Cu65
Zn66
Se78
Se82
Mo95
Mo98
-log(pvalue)
0
10
20
Age
Batch
Type
Lung
30

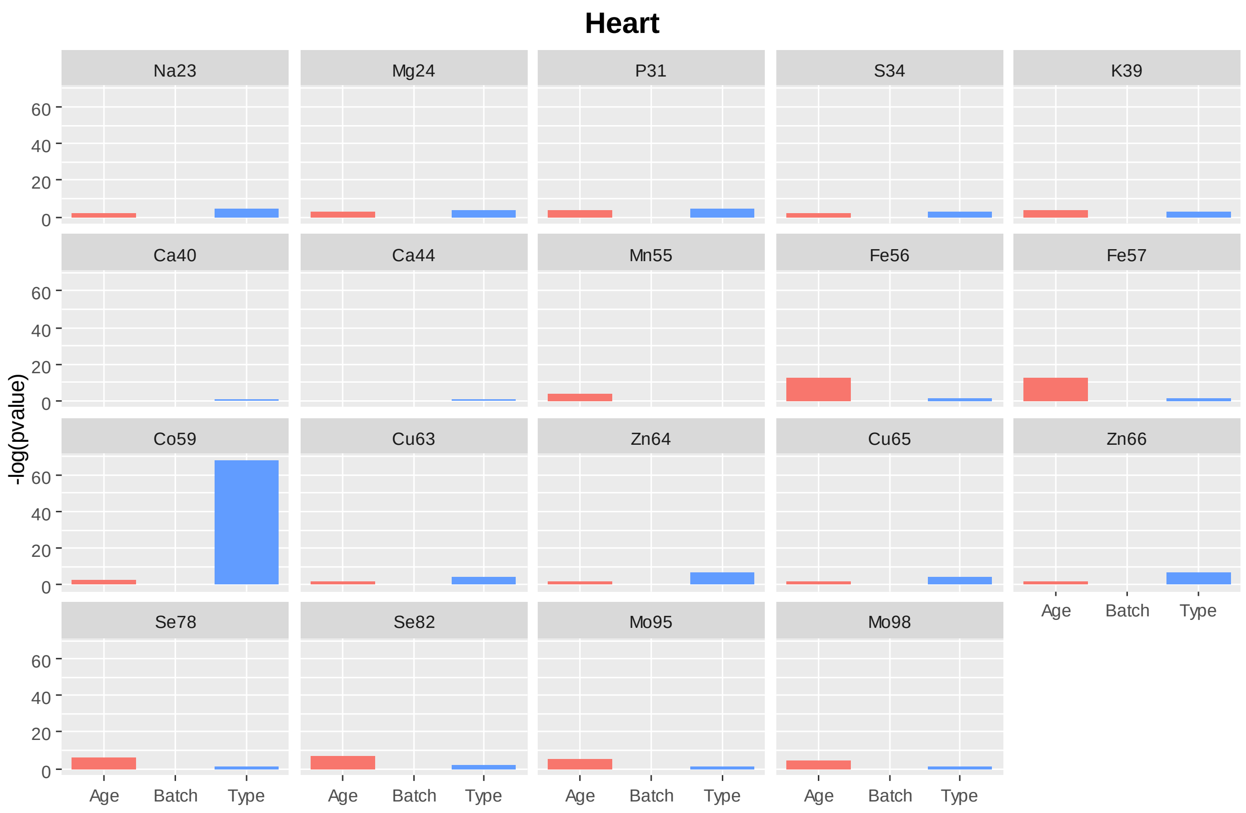
Heart
Na23
Mg24
P31
S34
K39
Ca40
Ca44
Mn55
Fe56
Fe57
Co59
Cu63
Zn64
Cu65
Zn66
Se78
Se82
Mo95
Mo98
-log(pvalue)
0
20
40
60
Age
Batch
Type

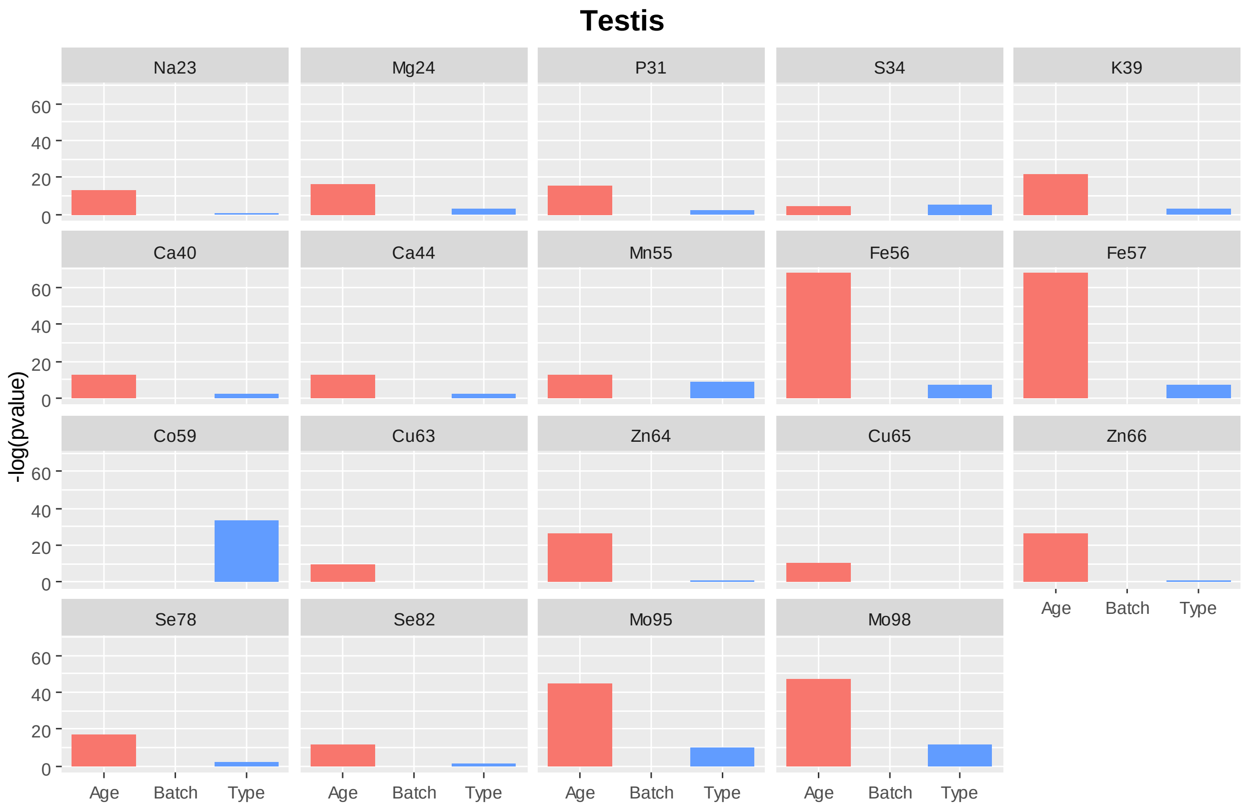
Testis
Na23
Mg24
P31
S34
K39
Ca40
Ca44
Mn55
Fe56
Fe57
Co59
Cu63
Zn64
Cu65
Zn66
Se78
Se82
Mo95
Mo98
-log(pvalue)
0
20
40
60
Age
Batch
Type

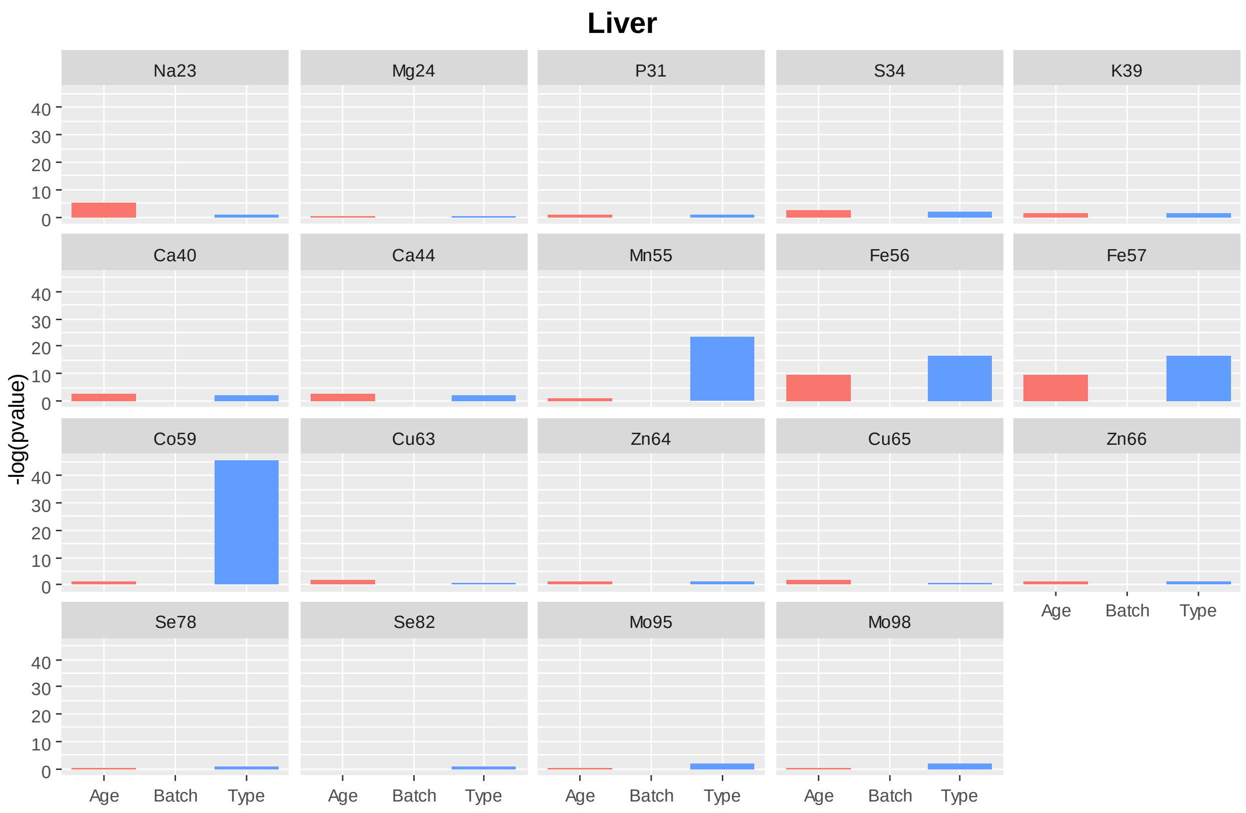
Liver
Na23
Mg24
P31
S34
K39
Ca40
Ca44
Mn55
Fe56
Fe57
Co59
Cu63
Zn64
Cu65
Zn66
Se78
Se82
Mo95
Mo98
-log(pvalue)
0
10
20
30
40
Age
Batch
Type

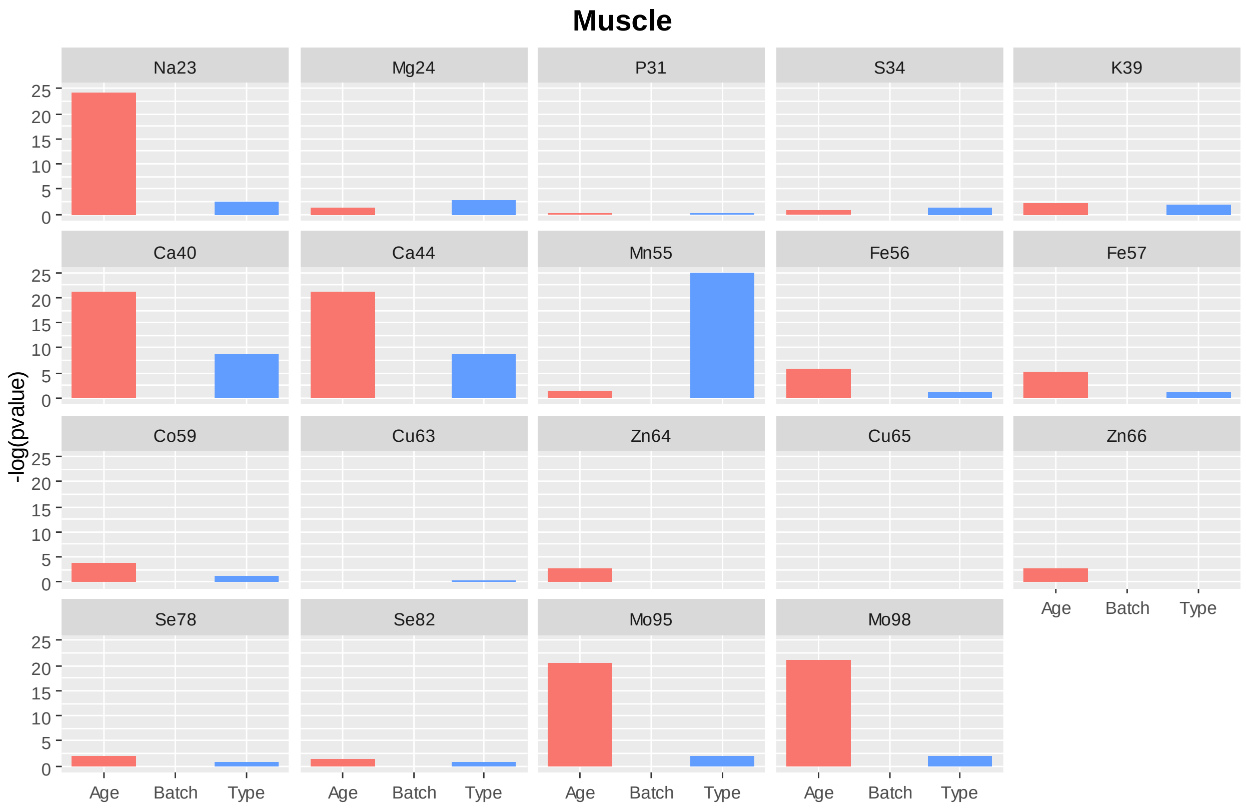
Muscle
Na23
Mg24
P31
S34
K39
Ca40
Ca44
Mn55
Fe56
Fe57
Co59
Cu63
Zn64
Cu65
Zn66
Se78
Se82
Mo95
Mo98
-log(pvalue)
0
5
10
15
20
25
Age
Batch
Type

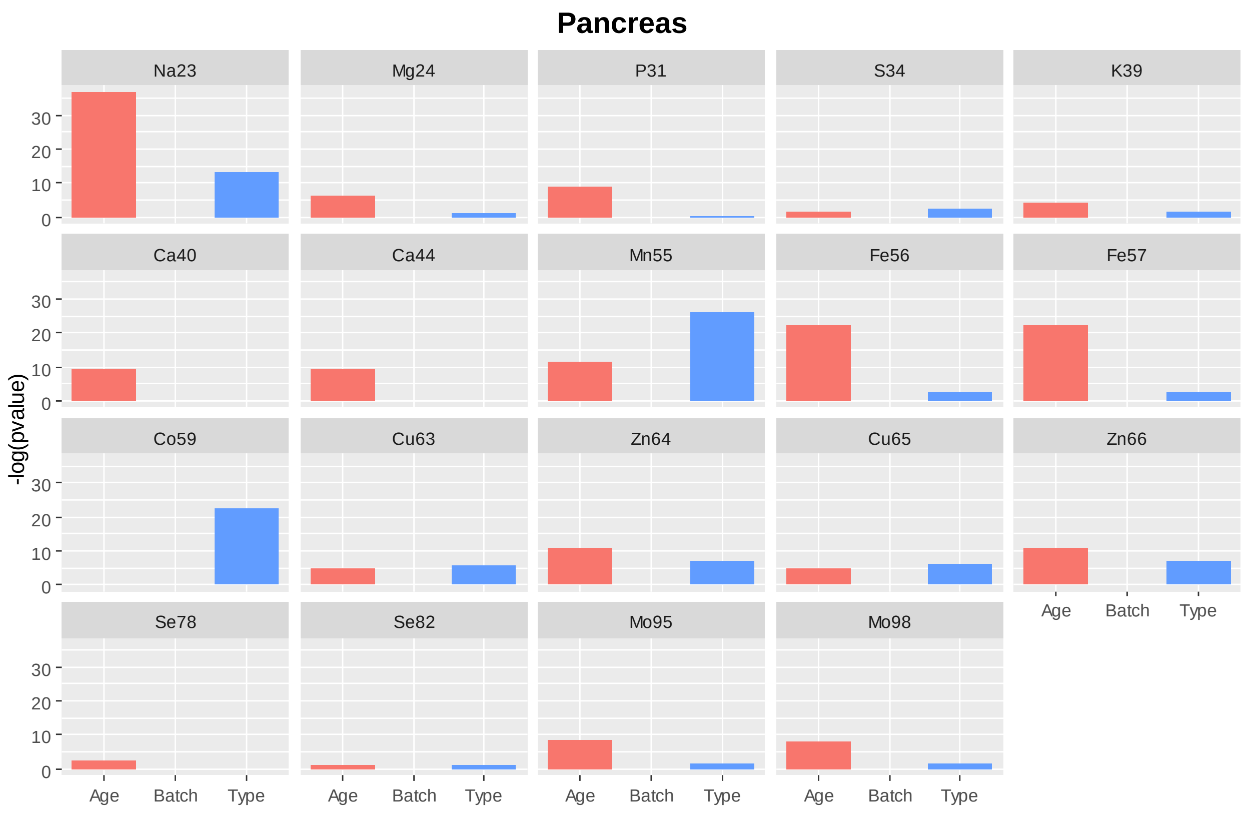
Pancreas
Na23
Mg24
P31
S34
K39
Ca40
Ca44
Mn55
Fe56
Fe57
Co59
Cu63
Zn64
Cu65
Zn66
Se78
Se82
Mo95
Mo98
-log(pvalue)
0
10
20
30
Age
Batch
Type

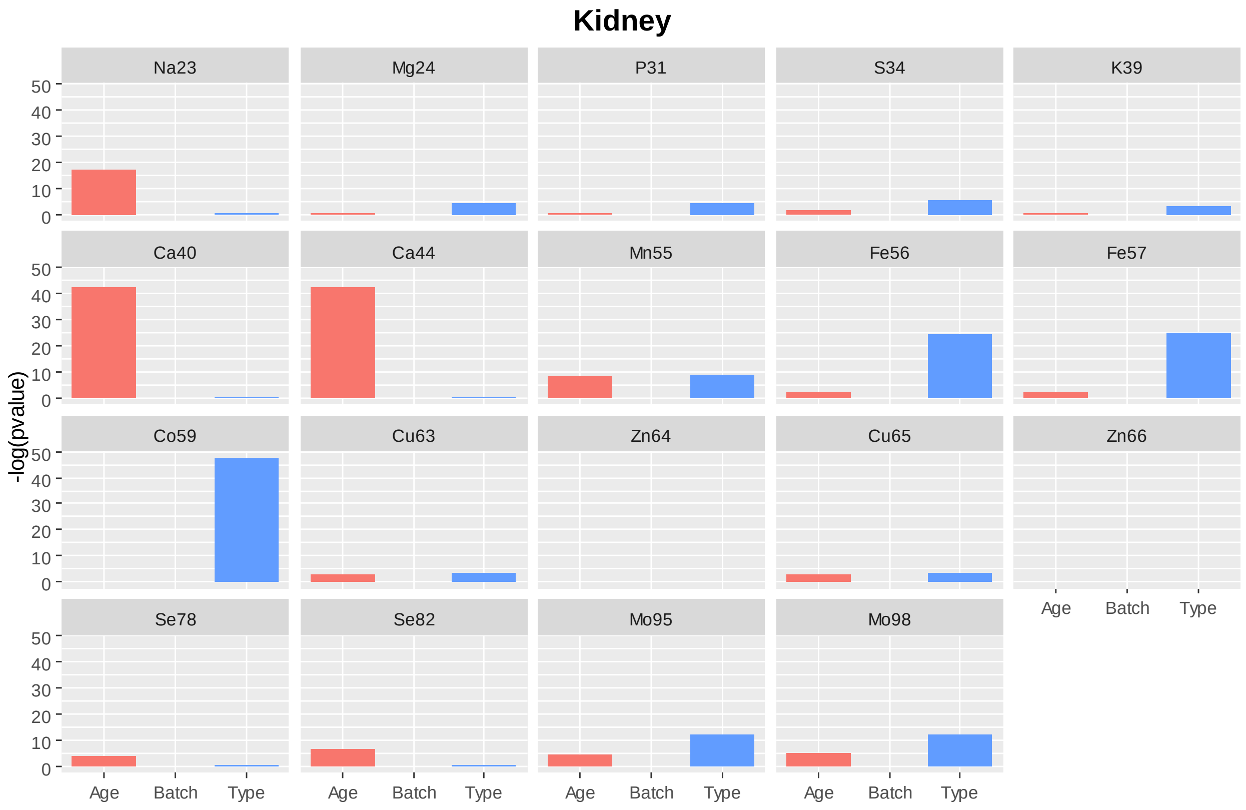
Kidney
Na23
Mg24
P31
S34
K39
Ca40
Ca44
Mn55
Fe56
Fe57
Co59
Cu63
Zn64
Cu65
Zn66
Se78
Se82
Mo95
Mo98
-log(pvalue)
0
10
20
30
40
50
Age
Batch
Type

Fig. S3

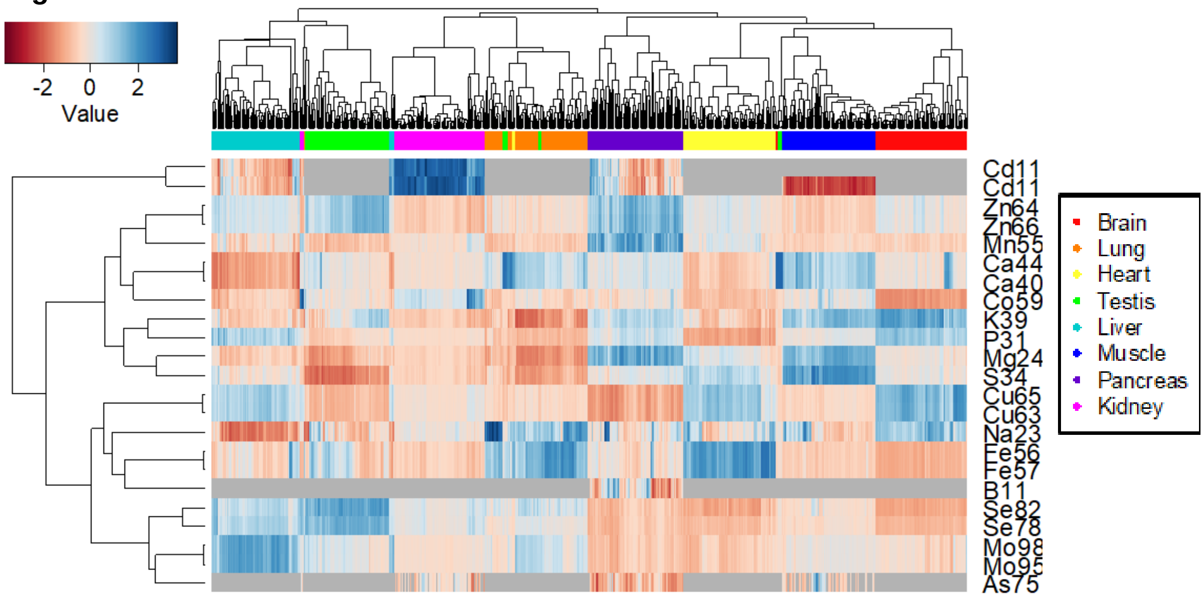
-2 0 2
Value
Cd111
Cd111
Zn64
Zn66
Mn55
Ca44
Ca40
Co59
K39
P31
Mg24
S34
Cu65
Cu63
Na23
Fe56
Fe57
B11
Se82
Se78
Mo98
Mo95
As75
Brain
Lung
Heart
Testis
Liver
Muscle
Pancreas
Kidney

Fig. S4

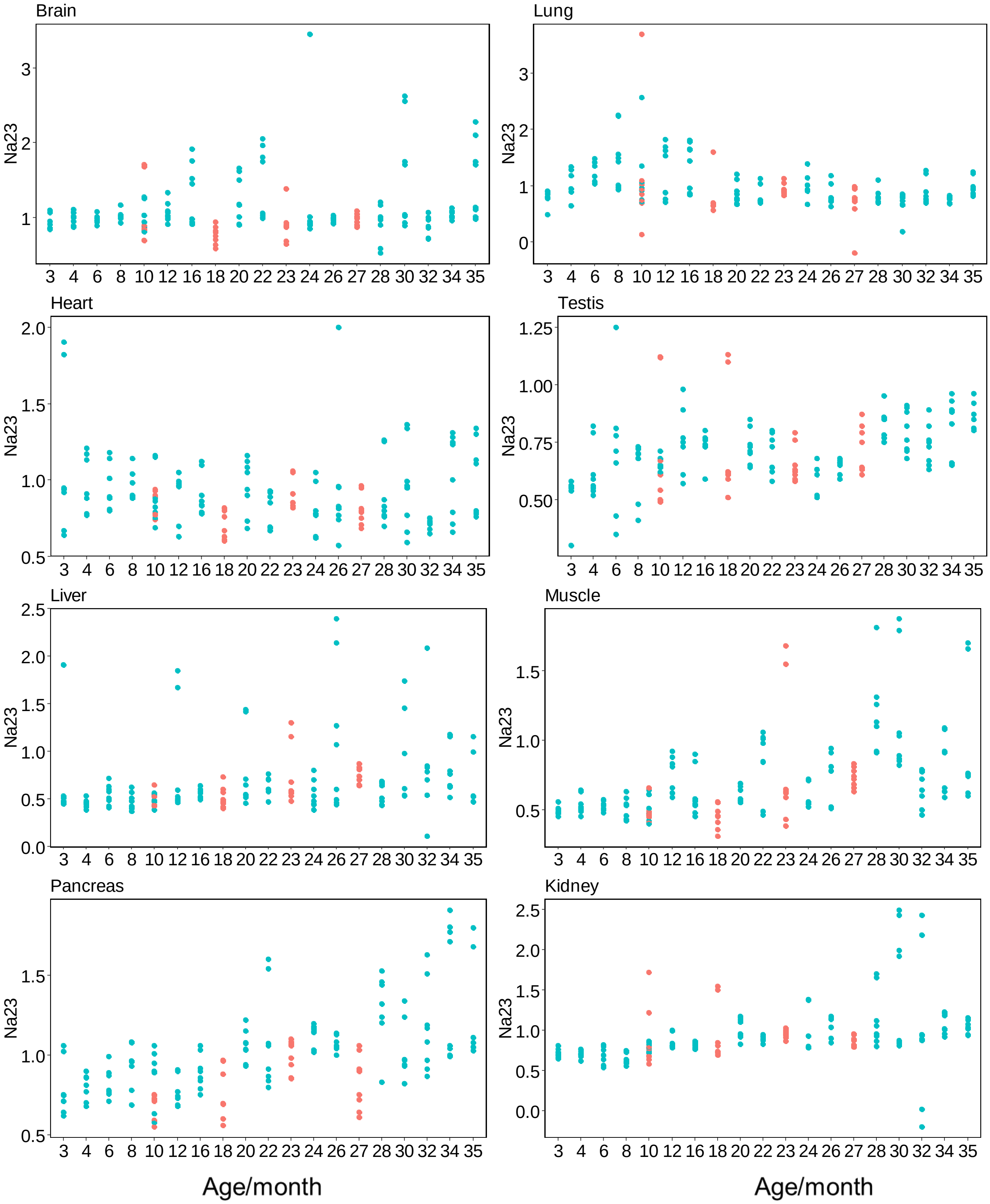

Brain
Lung
Heart
Testis
Liver
Muscle
Pancreas
Kidney
Na23
3 4 6 8 10 12 16 18 20 22 23 24 26 27 28 30 32 34 35
Age/month
Age/month

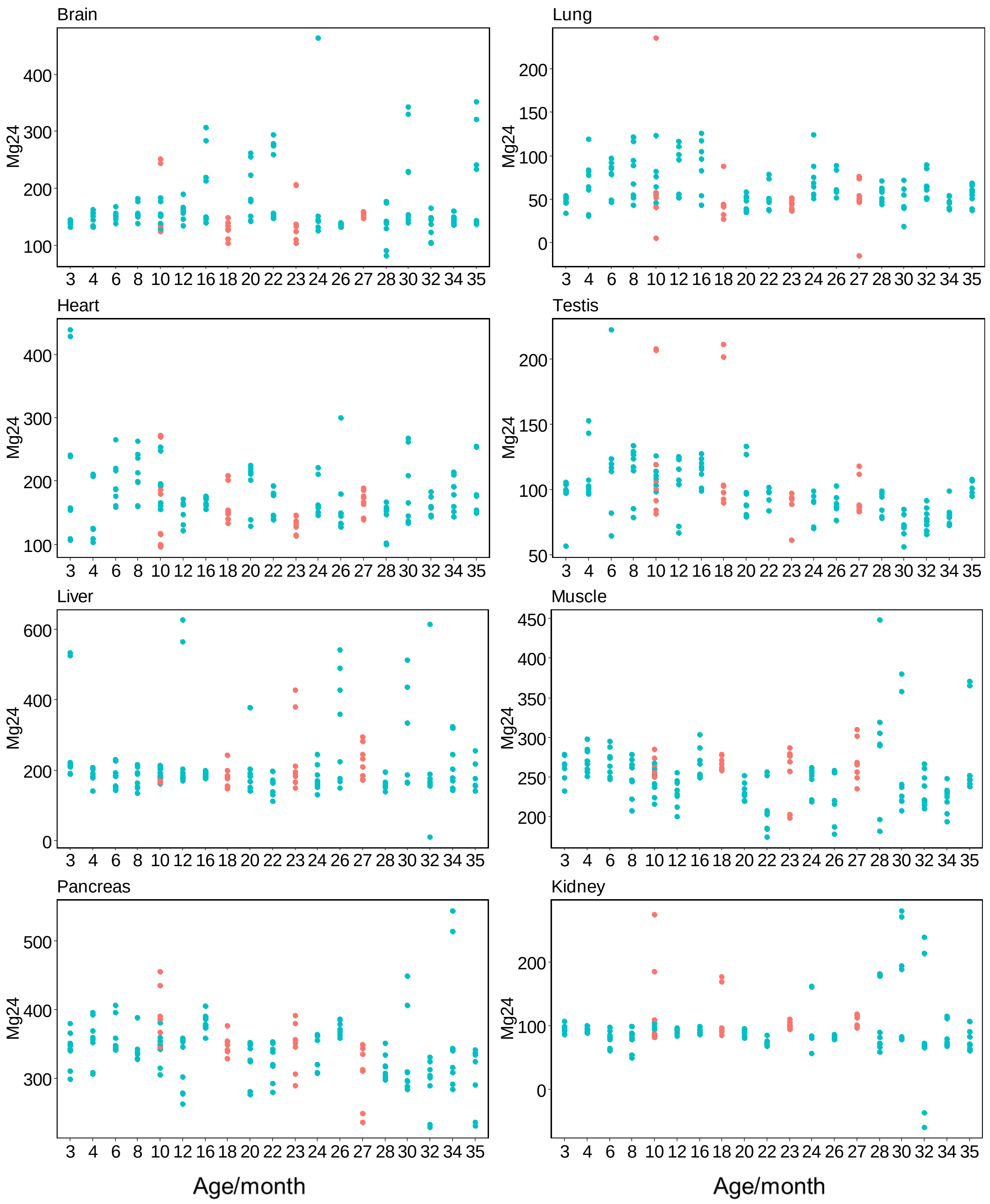
Brain
Lung
Heart
Testis
Liver
Muscle
Pancreas
Kidney
Mg24
3 4 6 8 10 12 16 18 20 22 23 24 26 27 28 30 32 34 35
Age/month

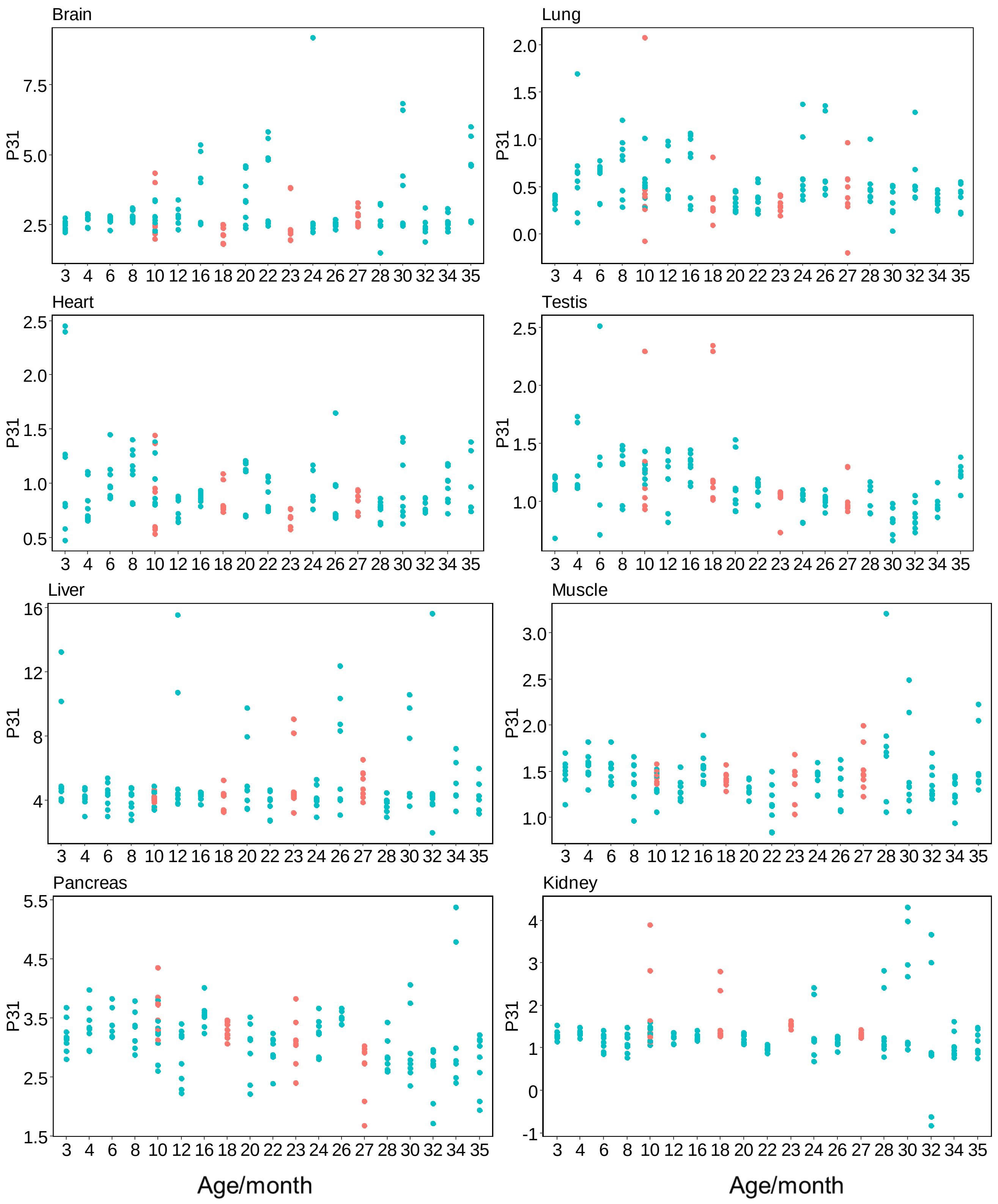
Brain
Lung
Heart
Testis
Liver
Muscle
Pancreas
Kidney
P31
Age/month
3 4 6 8 10 12 16 18 20 22 23 24 26 27 28 30 32 34 35

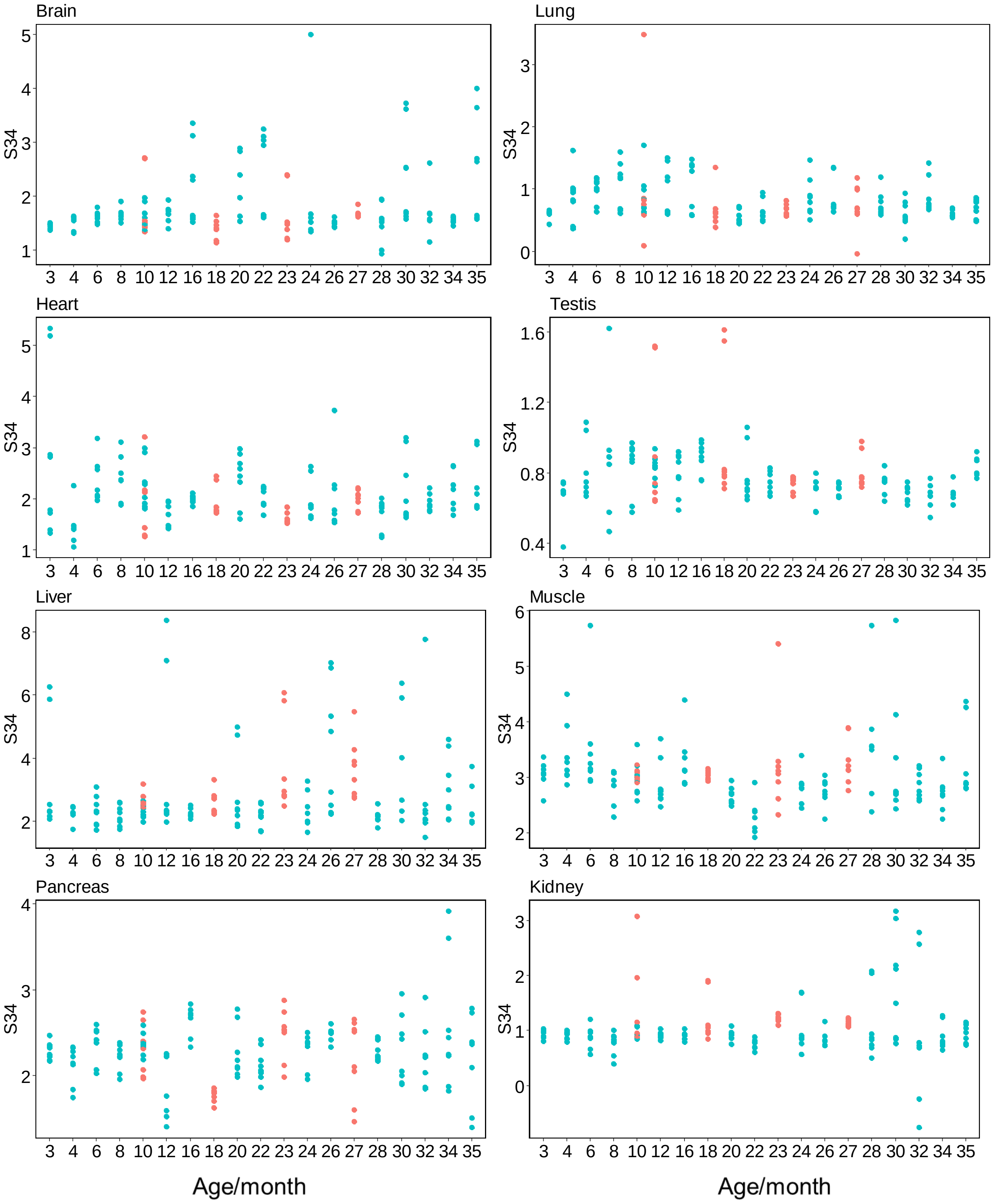
Brain
Lung
Heart
Testis
Liver
Muscle
Pancreas
Kidney
S34
3 4 6 8 10 12 16 18 20 22 23 24 26 27 28 30 32 34 35
Age/month
Age/month

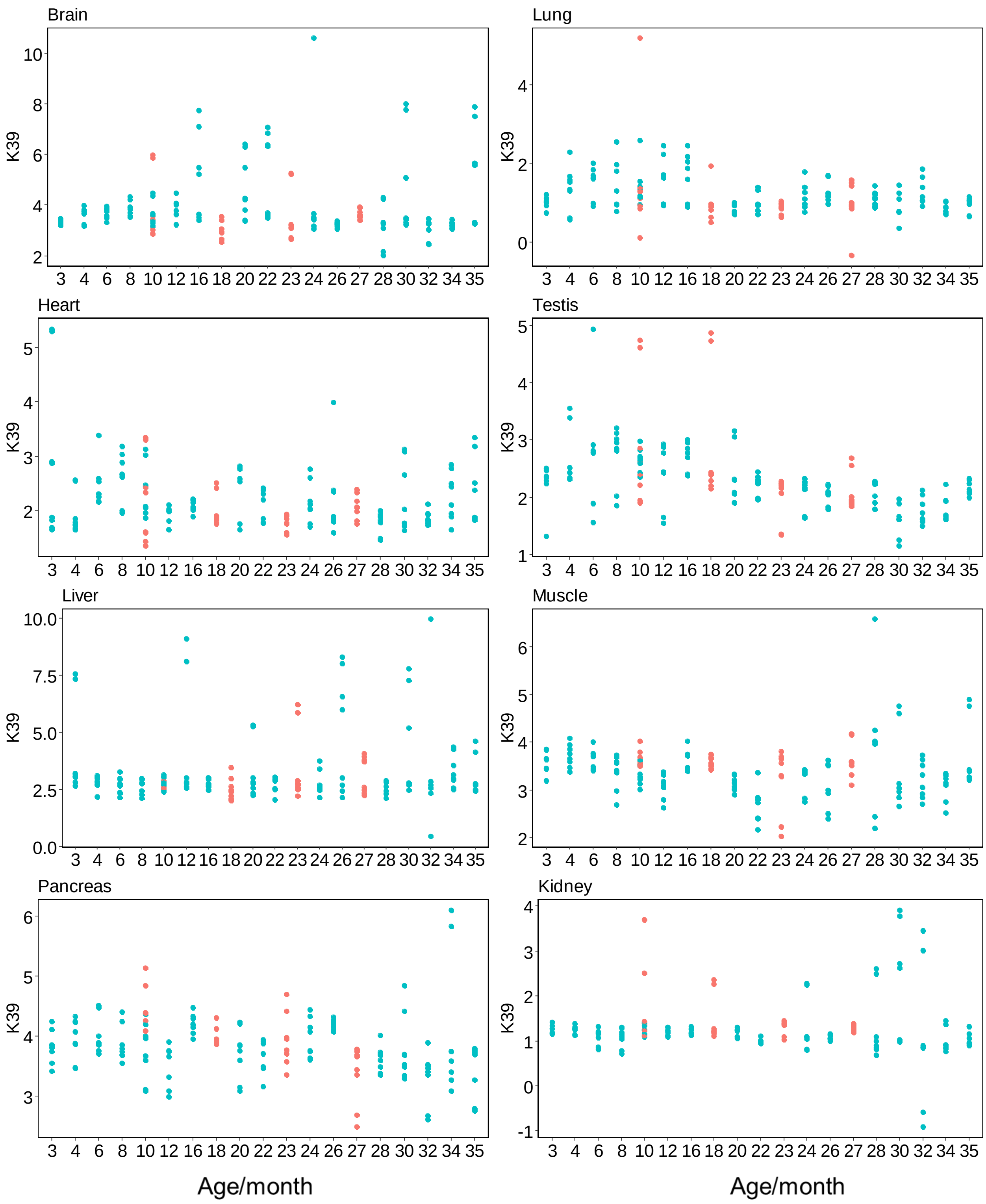
Brain
Lung
Heart
Testis
Liver
Muscle
Pancreas
Kidney
K39
3 4 6 8 10 12 16 18 20 22 23 24 26 27 28 30 32 34 35
Age/month

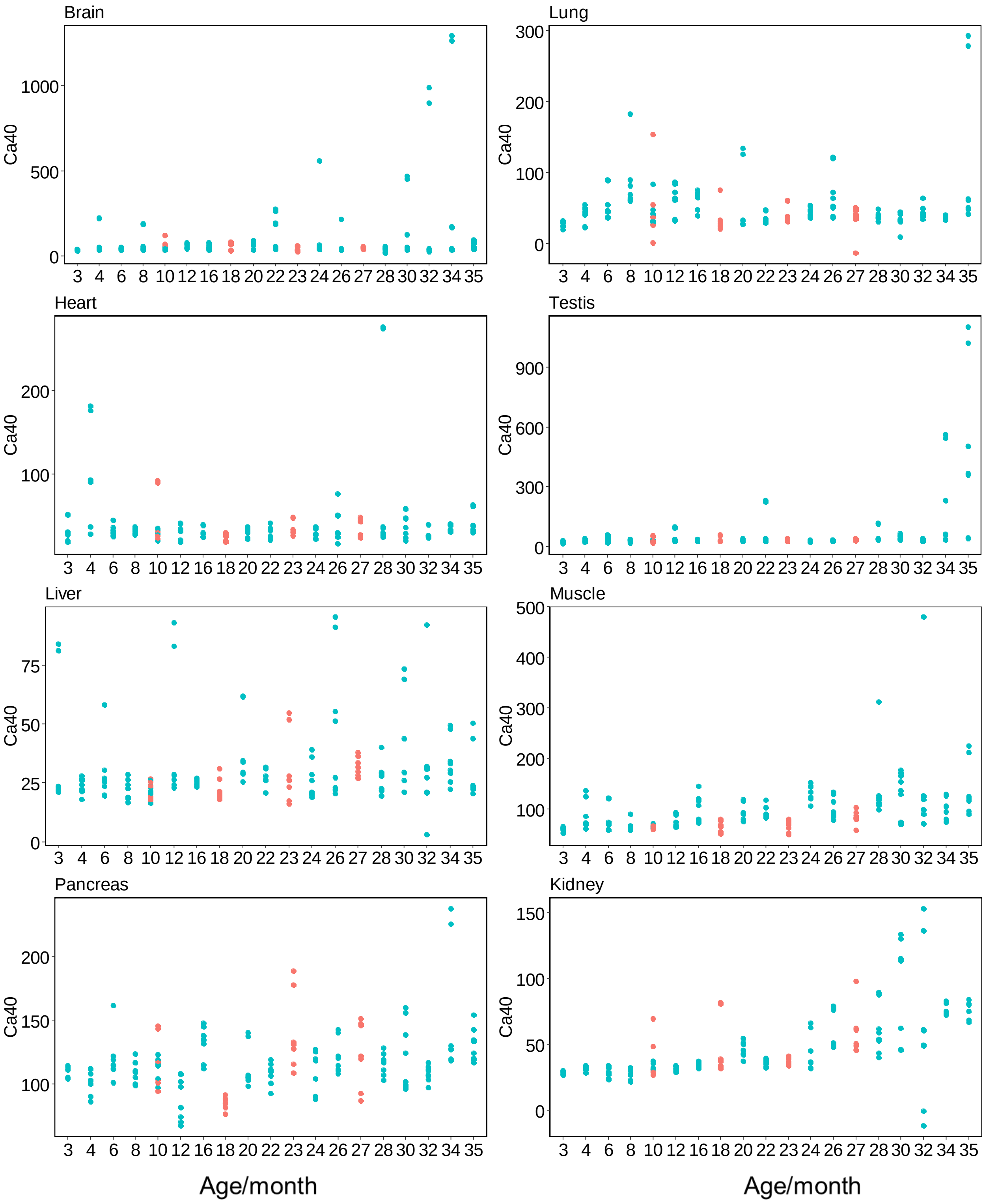

Brain
Lung
Heart
Testis
Liver
Muscle
Pancreas
Kidney
Ca40
Age/month
3 4 6 8 10 12 16 18 20 22 23 24 26 27 28 30 32 34 35

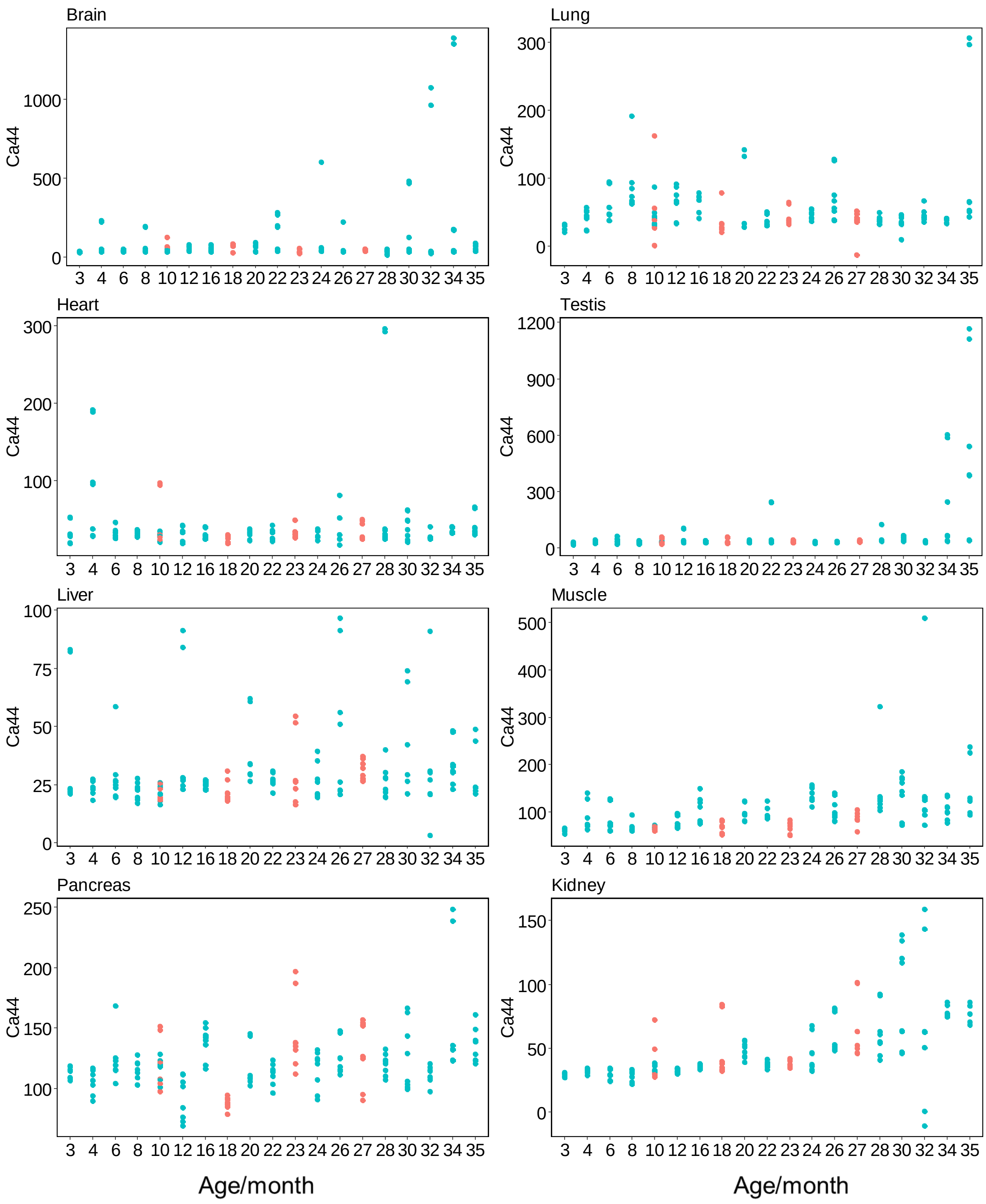
Brain
Lung
Heart
Testis
Liver
Muscle
Pancreas
Kidney
Ca44
Age/month
3 4 6 8 10 12 16 18 20 22 23 24 26 27 28 30 32 34 35

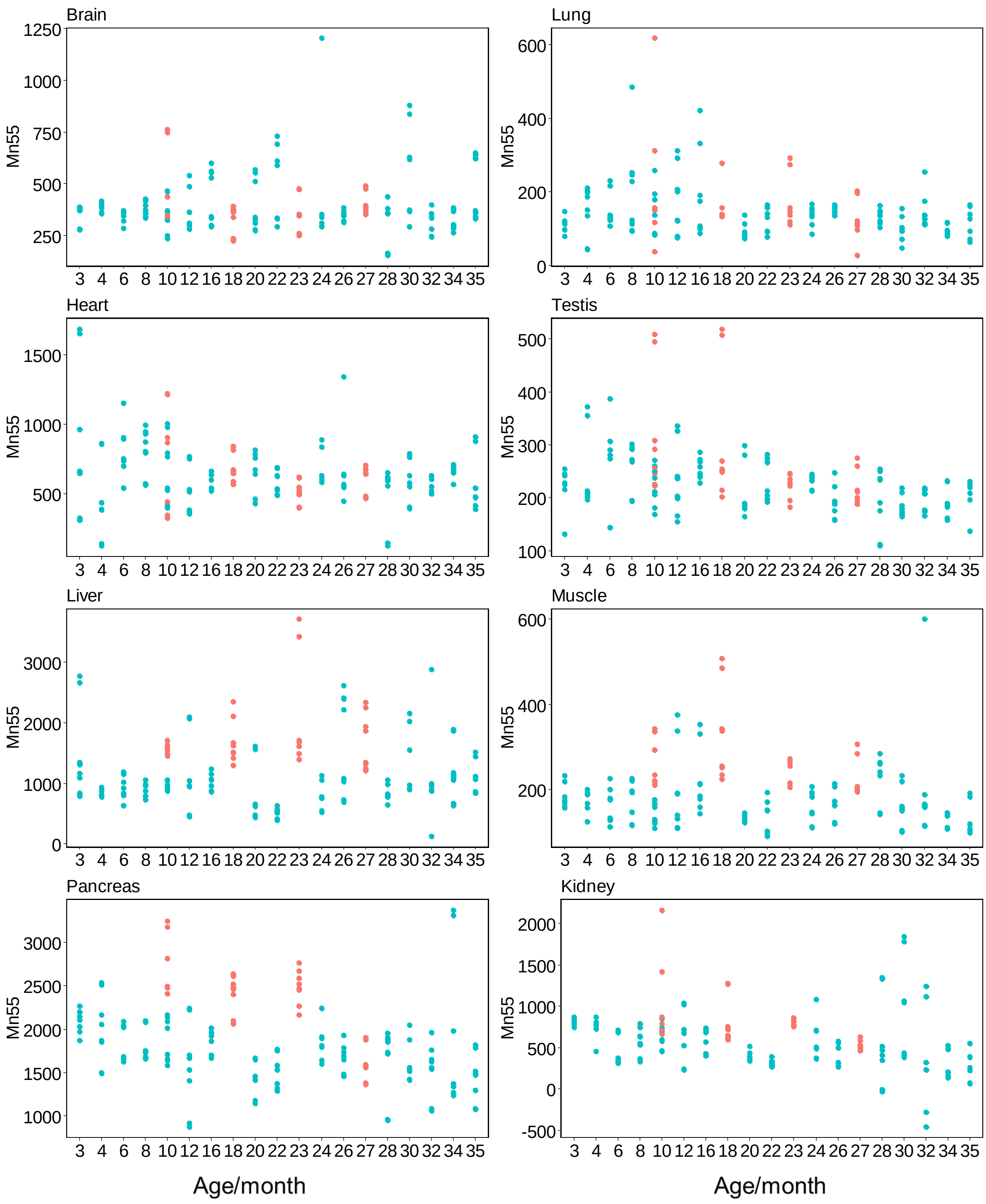

Brain
Lung
Heart
Testis
Liver
Muscle
Pancreas
Kidney
Mn55
3 4 6 8 10 12 16 18 20 22 23 24 26 27 28 30 32 34 35
Age/month

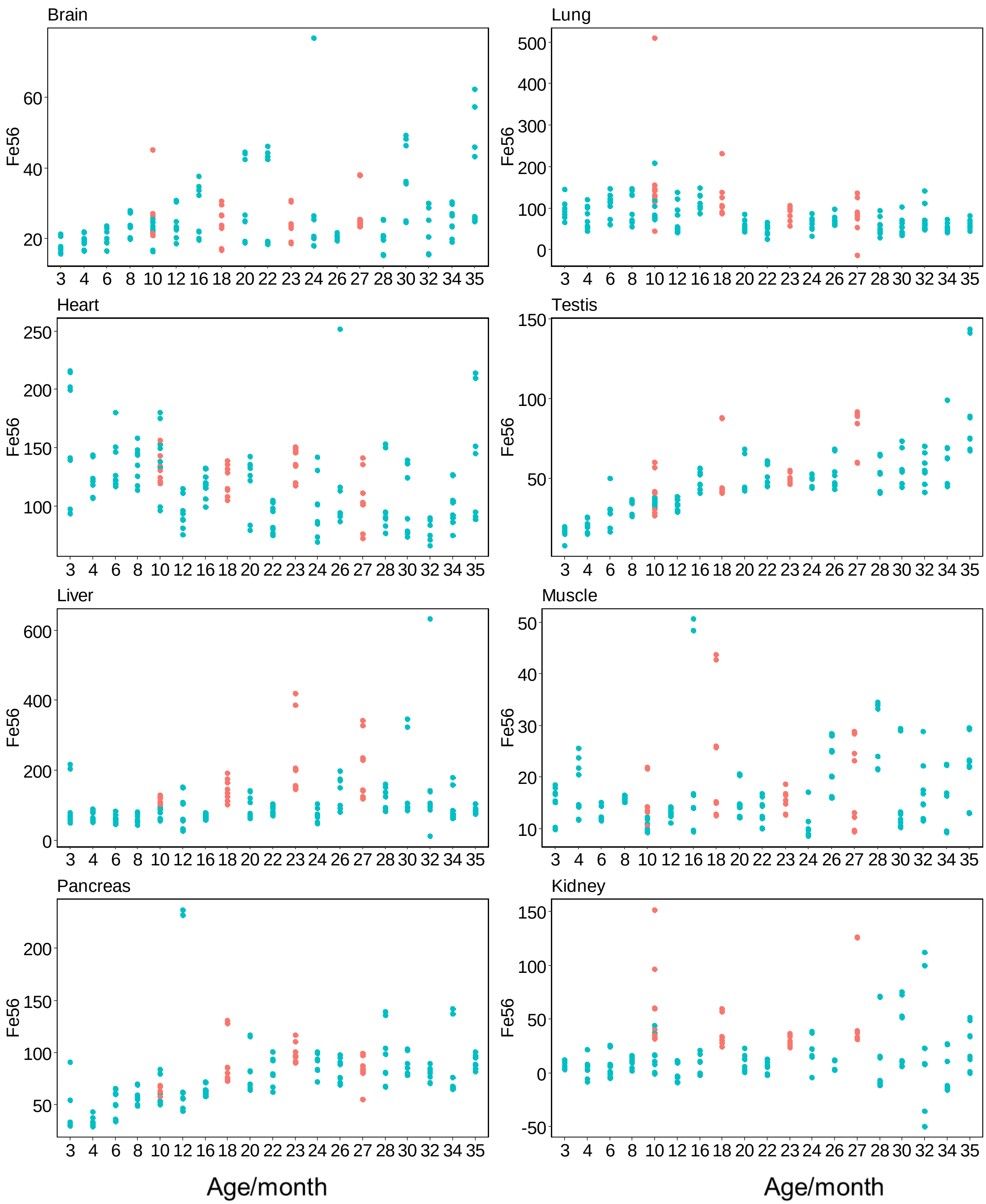
Brain
Lung
Heart
Testis
Liver
Muscle
Pancreas
Kidney
Fe56
Age/month
3 4 6 8 10 12 16 18 20 22 23 24 26 27 28 30 32 34 35

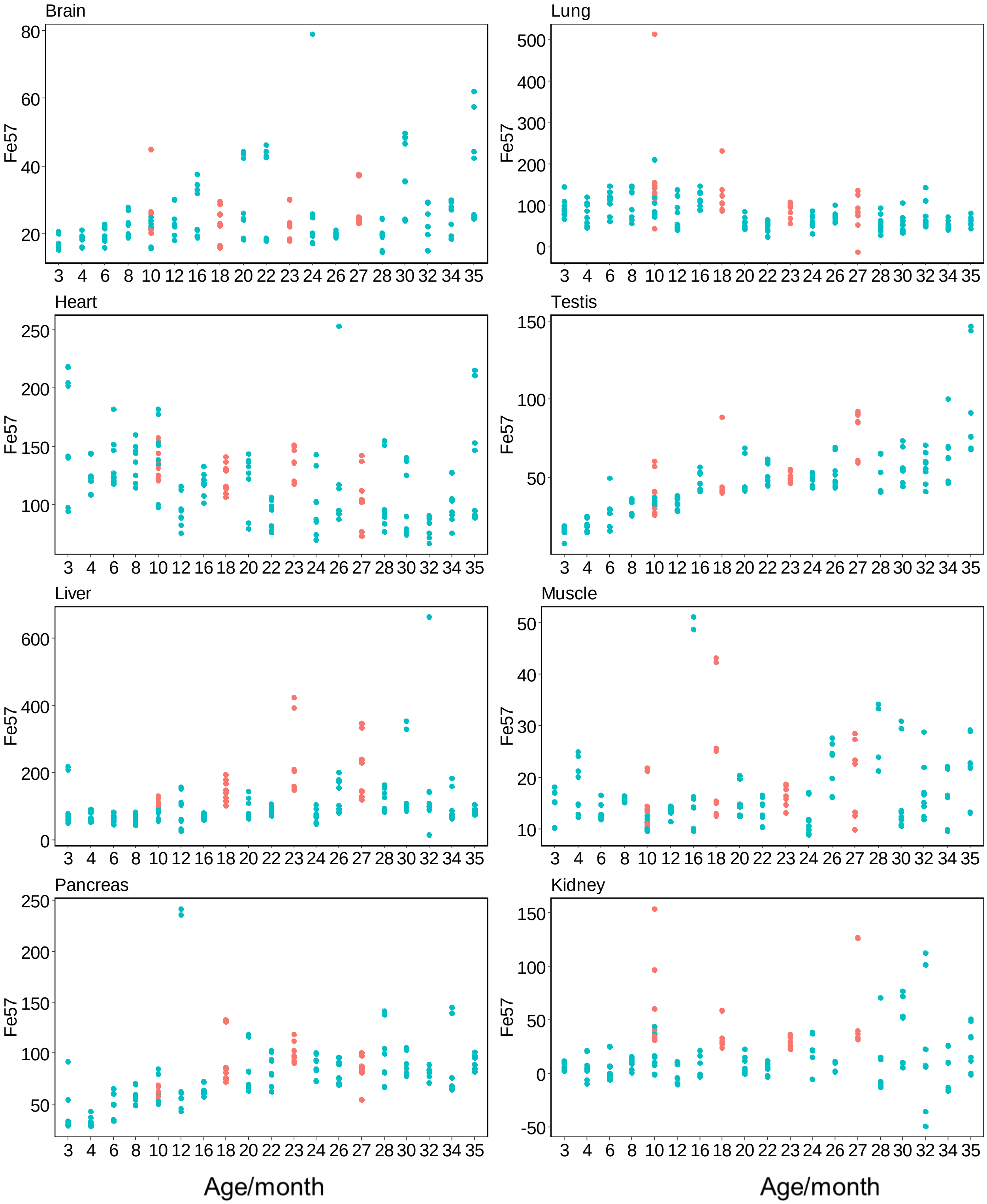
Brain
Lung
Heart
Testis
Liver
Muscle
Pancreas
Kidney
Fe57
3 4 6 8 10 12 16 18 20 22 23 24 26 27 28 30 32 34 35
Age/month
Age/month

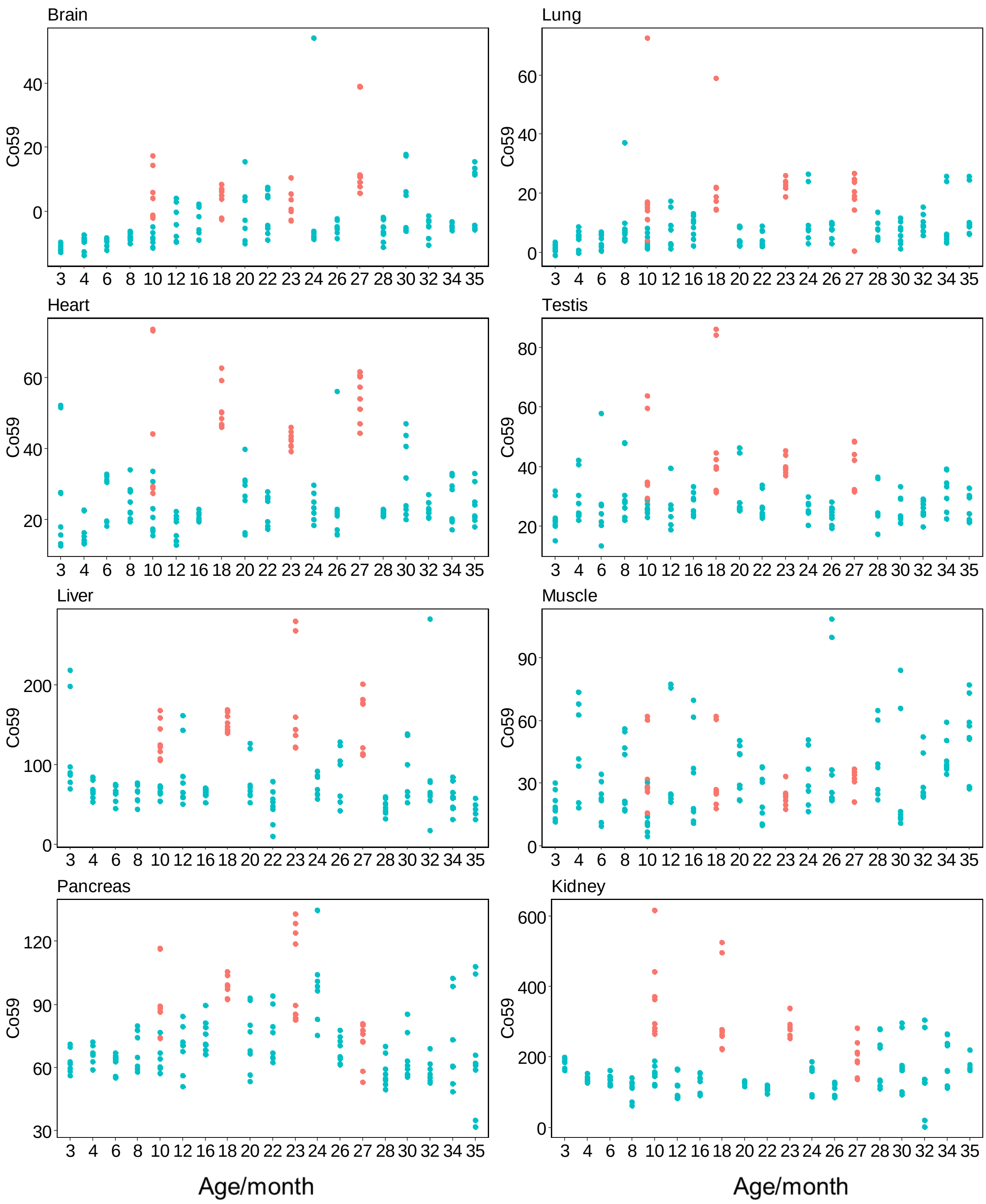

Brain
Lung
Heart
Testis
Liver
Muscle
Pancreas
Kidney
Co59
3 4 6 8 10 12 16 18 20 22 23 24 26 27 28 30 32 34 35
Age/month

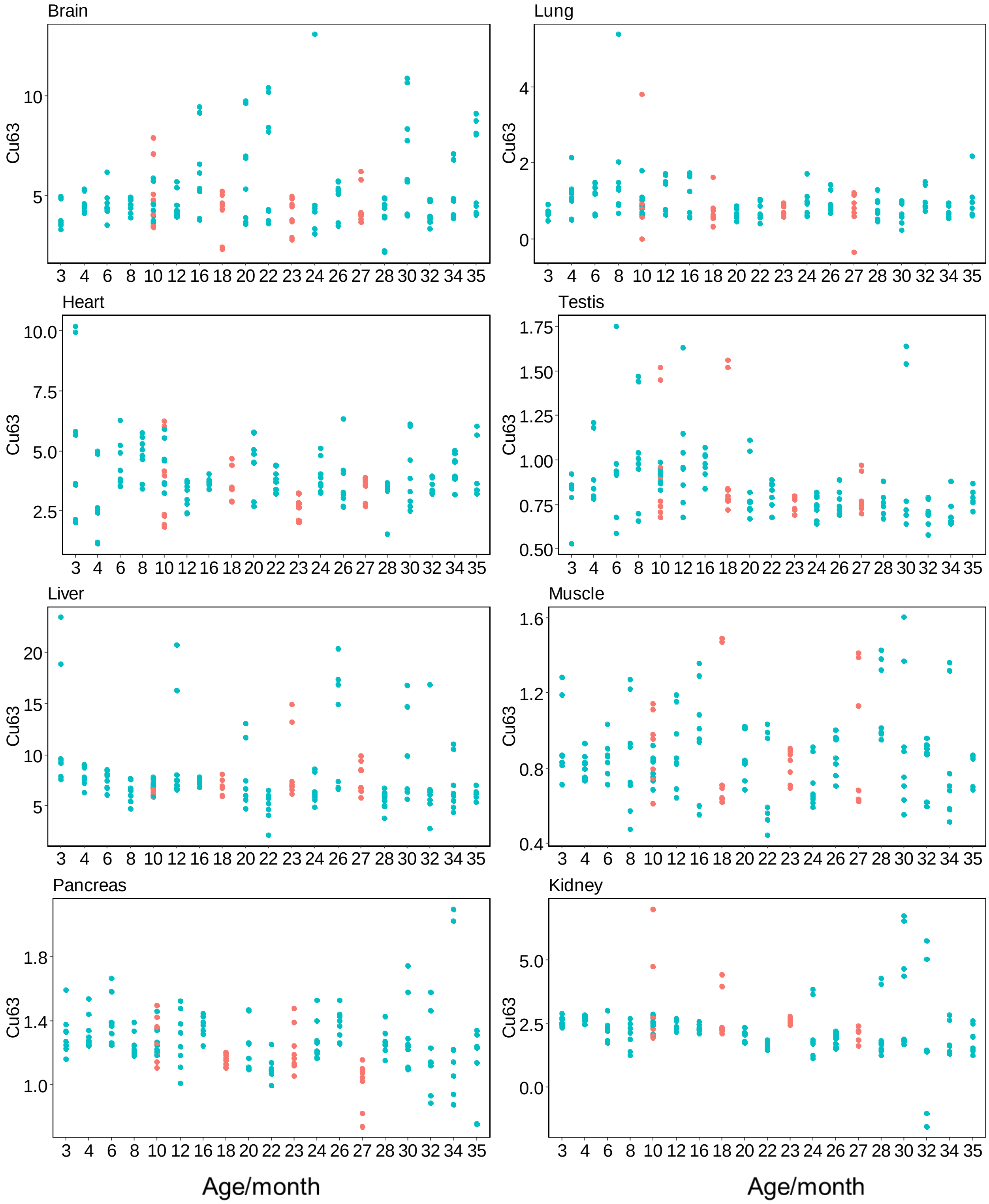

Brain
Lung
Heart
Testis
Liver
Muscle
Pancreas
Kidney
Cu63
3 4 6 8 10 12 16 18 20 22 23 24 26 27 28 30 32 34 35
Age/month

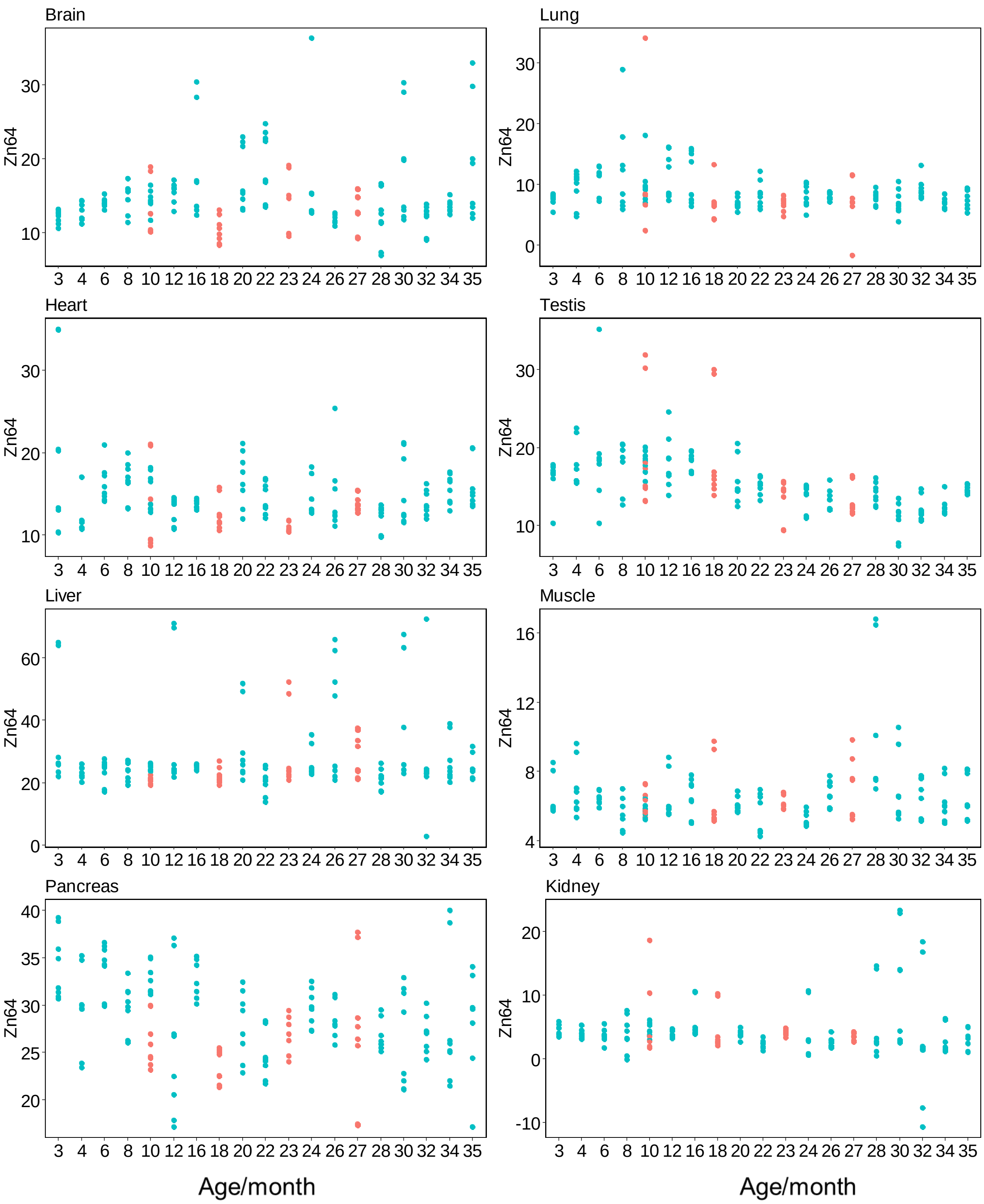
Brain
Lung
Heart
Testis
Liver
Muscle
Pancreas
Kidney
Zn64
3 4 6 8 10 12 16 18 20 22 23 24 26 27 28 30 32 34 35
Age/month

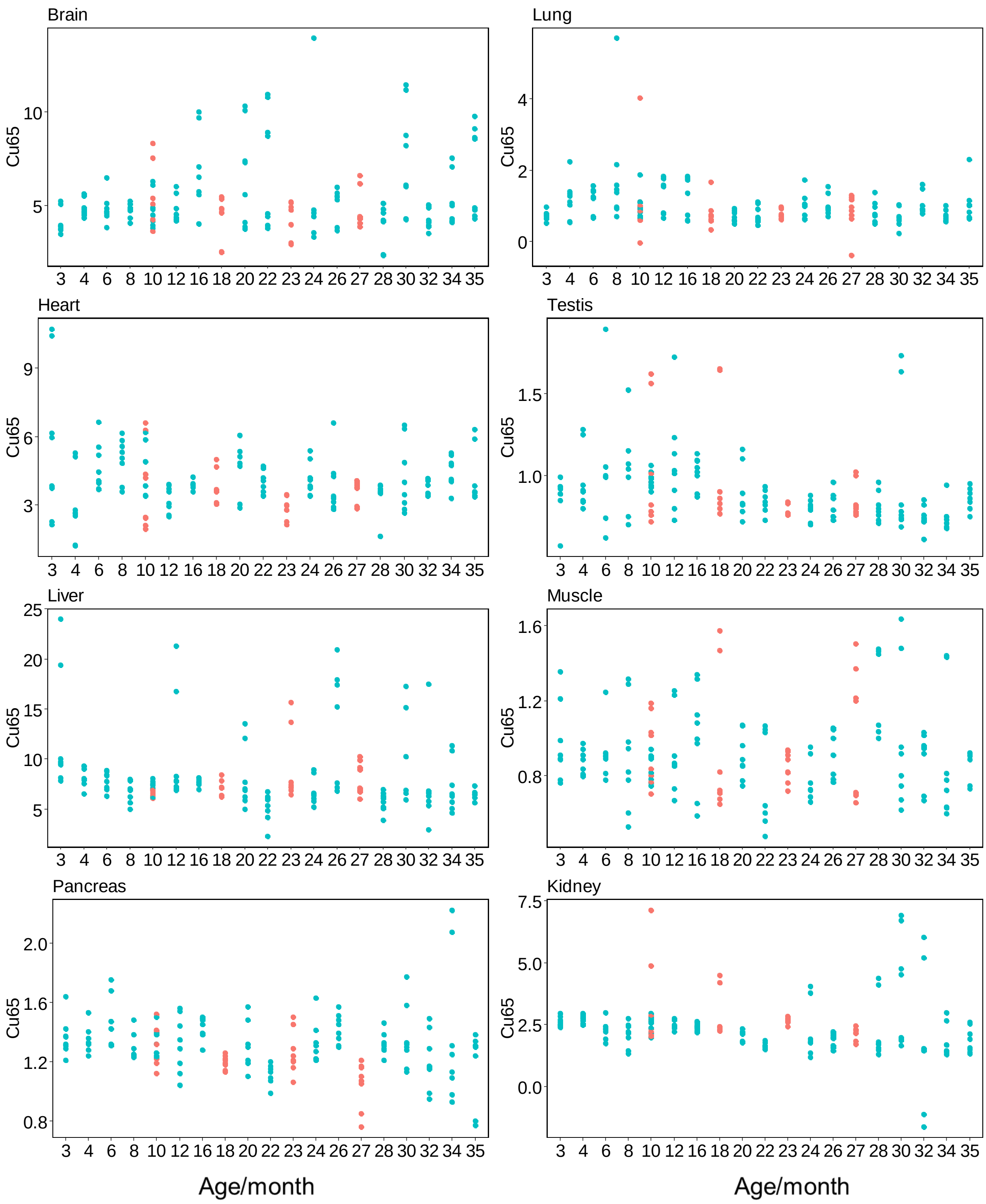
Brain
Lung
Heart
Testis
Liver
Muscle
Pancreas
Kidney
Cu65
3 4 6 8 10 12 16 18 20 22 23 24 26 27 28 30 32 34 35
Age/month

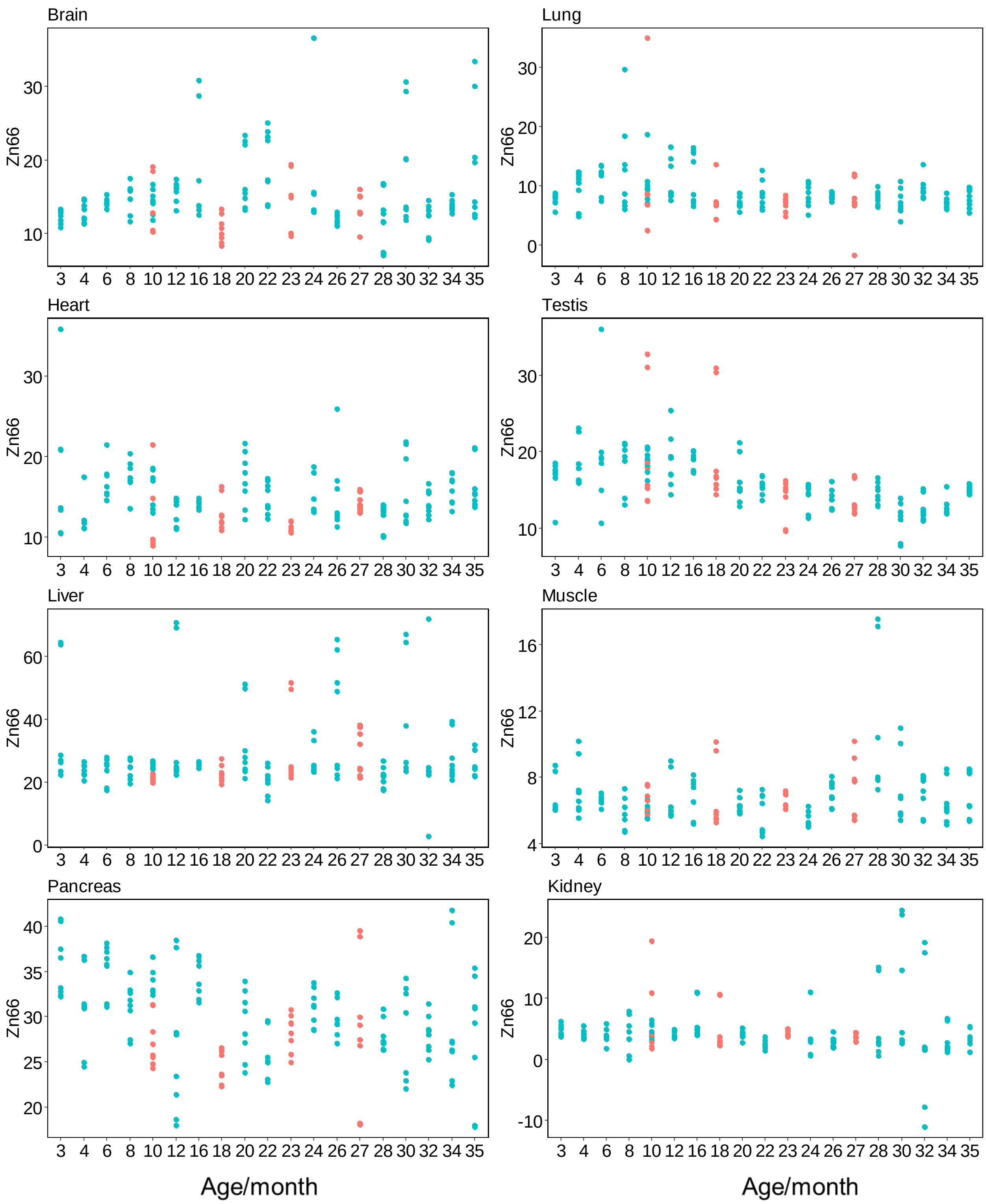
Brain
Lung
Heart
Testis
Liver
Muscle
Pancreas
Kidney
Zn66
3 4 6 8 10 12 16 18 20 22 23 24 26 27 28 30 32 34 35
Age/month

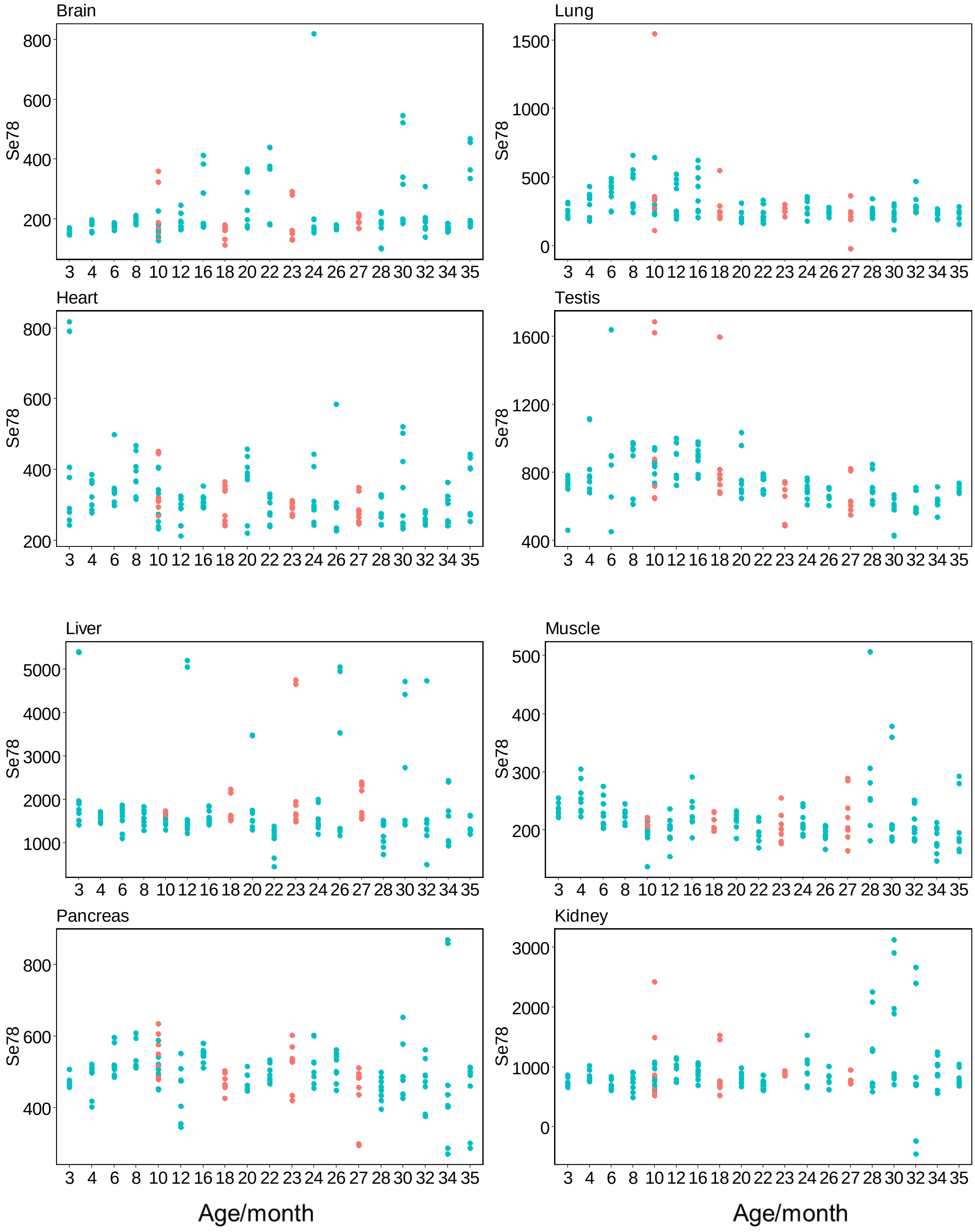

Brain
Lung
Heart
Testis
Liver
Muscle
Pancreas
Kidney
Se78
Age/month
3 4 6 8 10 12 16 18 20 22 23 24 26 27 28 30 32 34 35

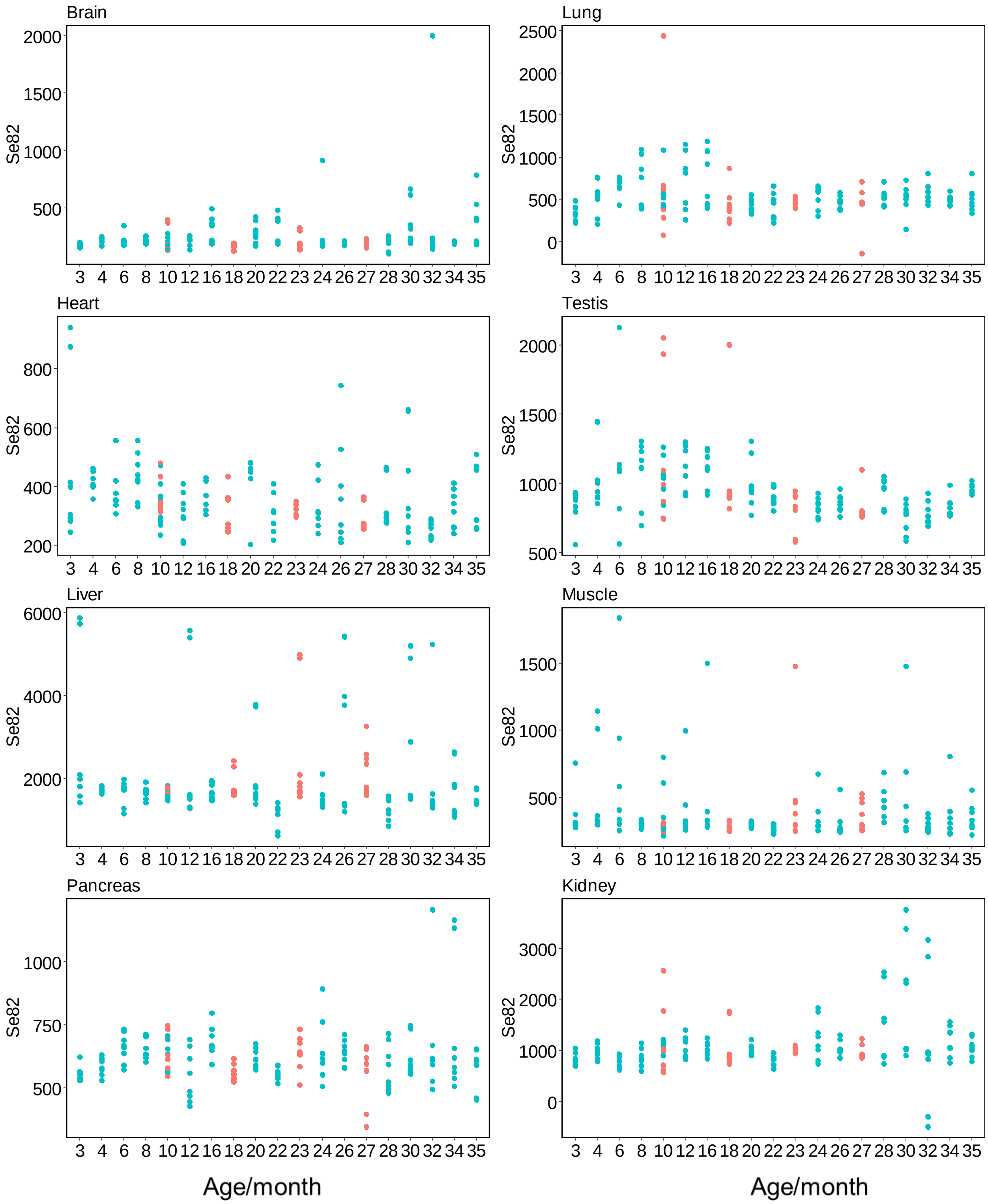

Brain
Lung
Heart
Testis
Liver
Muscle
Pancreas
Kidney
Se82
Age/month
Age/month

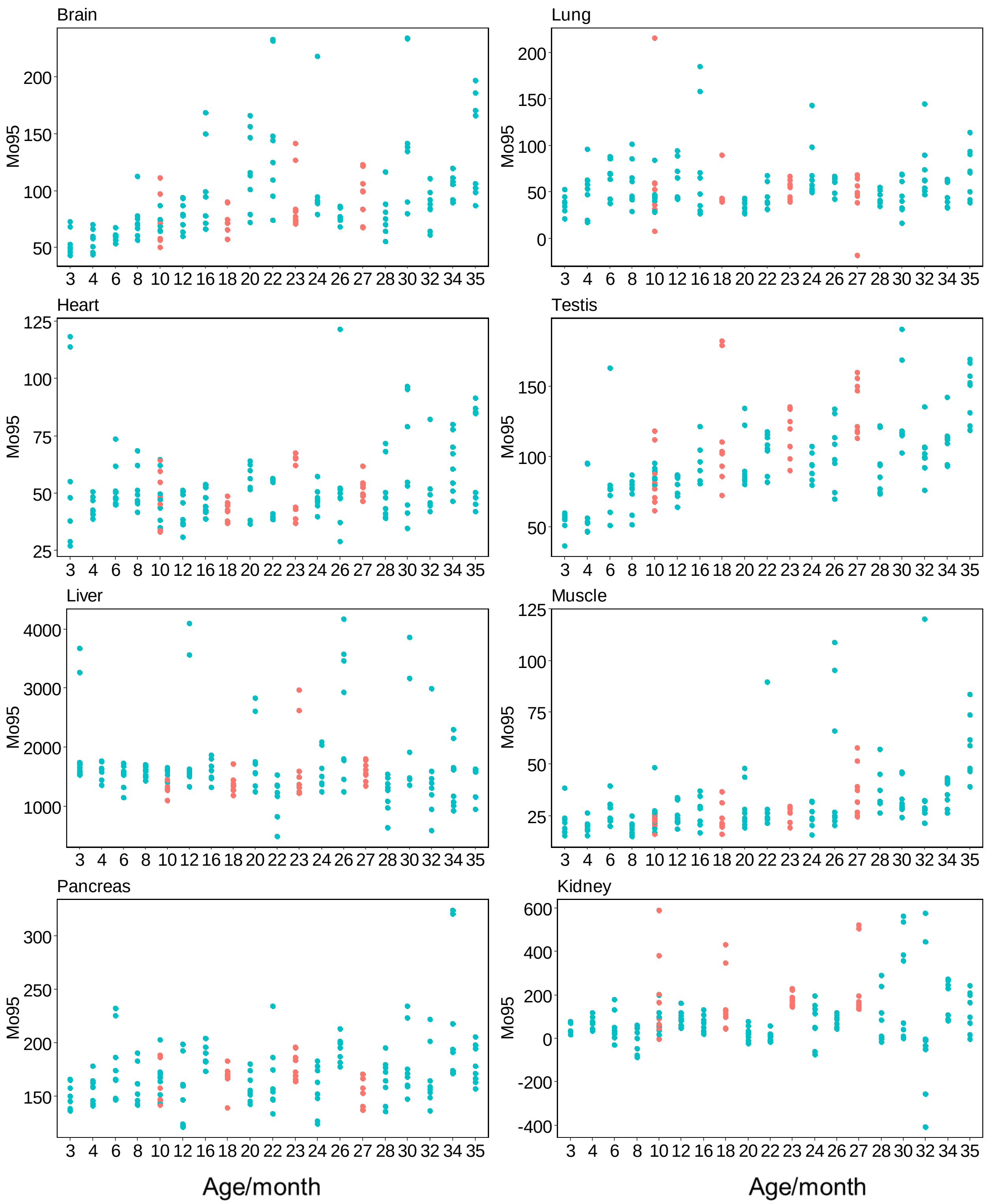

Brain
Lung
Heart
Testis
Liver
Muscle
Pancreas
Kidney
Mo95
3 4 6 8 10 12 16 18 20 22 23 24 26 27 28 30 32 34 35
Age/month

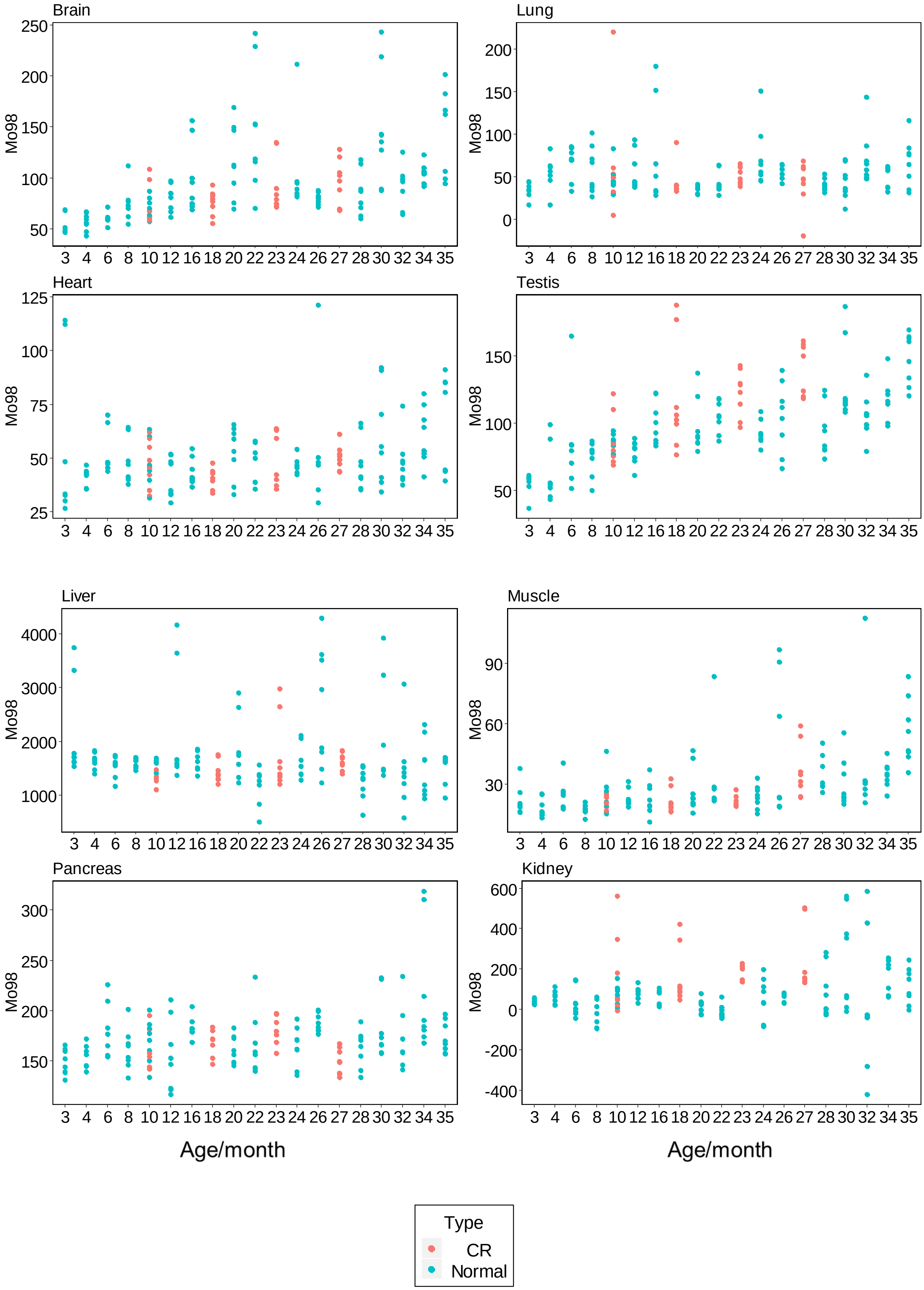
Brain
Lung
Heart
Testis
Liver
Muscle
Pancreas
Kidney
Mo98
Age/month
Type
CR
Normal

Fig. S5

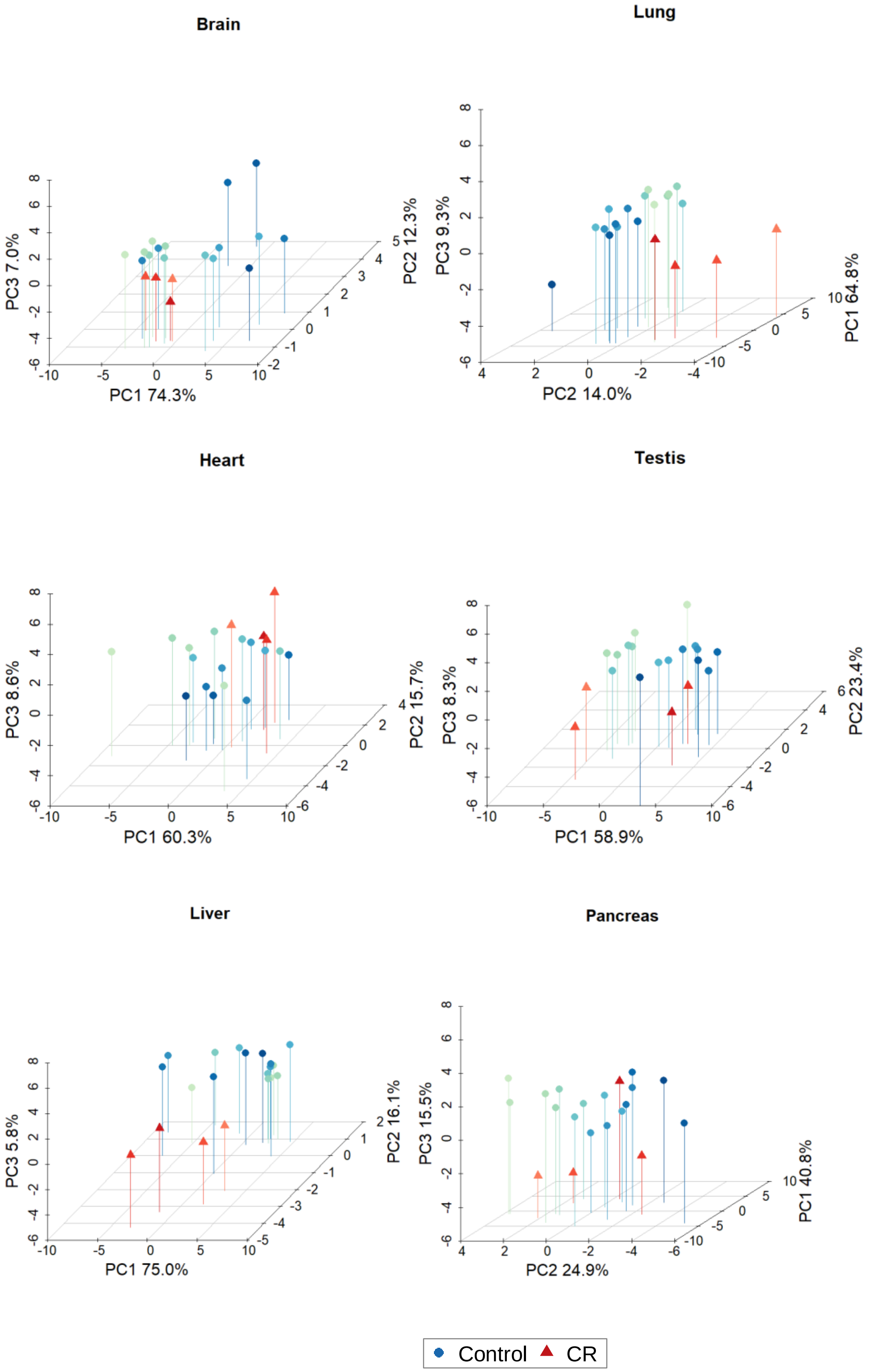
Brain
PC3 7.0%
PC1 74.3%
PC2 12.3%
Lung
PC3 9.3%
PC2 14.0%
PC1 64.8%
Heart
PC3 8.6%
PC1 60.3%
PC2 15.7%
Testis
PC3 8.3%
PC1 58.9%
PC2 23.4%
Liver
PC3 5.8%
PC1 75.0%
PC2 16.1%
Pancreas
PC3 15.5%
PC2 24.9%
PC1 40.8%
Control
CR

Fig. S6

A

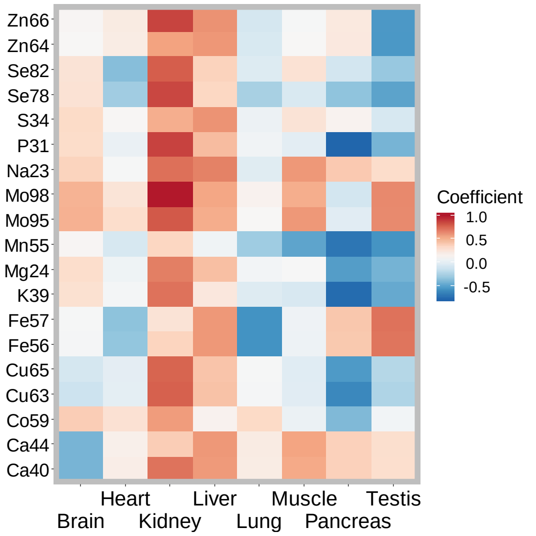
Zn66
Zn64
Se82
Se78
S34
P31
Na23
Mo98
Mo95
Mn55
Mg24
K39
Fe57
Fe56
Cu65
Cu63
Co59
Ca44
Ca40
Heart Liver Muscle Testis
Brain Kidney Lung Pancreas
Coefficient
1.0
0.5
0.0
-0.5

B

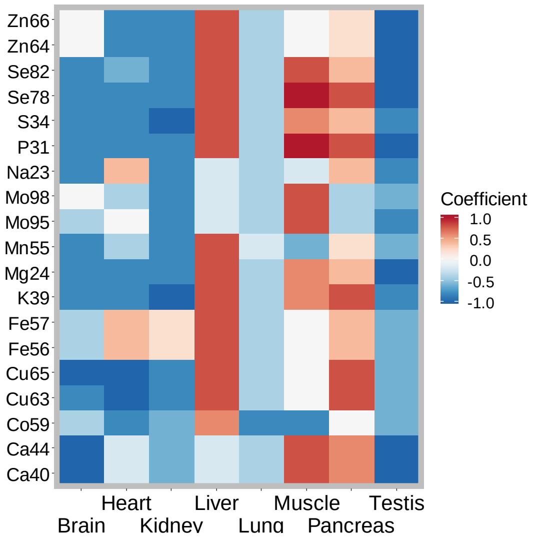
Zn66
Zn64
Se82
Se78
S34
P31
Na23
Mo98
Mo95
Mn55
Mg24
K39
Fe57
Fe56
Cu65
Cu63
Co59
Ca44
Ca40
Heart Liver Muscle Testis
Brain Kidney Lung Pancreas
Coefficient
1.0
0.5
0.0
-0.5
-1.0

C

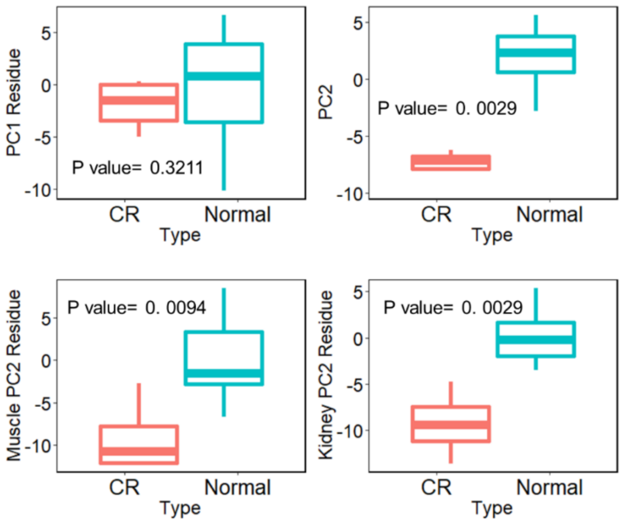
PC1 Residue
P value= 0.3211
PC2
P value= 0. 0029
Muscle PC2 Residue
P value= 0. 0094
Kidney PC2 Residue
P value= 0. 0029
CR Normal
Type
